# Allosteric control of Ubp6 and the proteasome via a bidirectional switch

**DOI:** 10.1038/s41467-022-28186-y

**Published:** 2022-02-11

**Authors:** Ka Ying Sharon Hung, Sven Klumpe, Markus R. Eisele, Suzanne Elsasser, Geng Tian, Shuangwu Sun, Jamie A. Moroco, Tat Cheung Cheng, Tapan Joshi, Timo Seibel, Duco Van Dalen, Xin-Hua Feng, Ying Lu, Huib Ovaa, John R. Engen, Byung-Hoon Lee, Till Rudack, Eri Sakata, Daniel Finley

**Affiliations:** 1 Department of Cell Biology, Harvard Medical School, Boston, MA 02115 USA; 2 Department of Molecular Structural Biology, Max Planck Institute of Biochemistry, 82152 Martinsried, Germany; 3 Life Sciences Institute (LSI), Zhejiang University, Hangzhou, 310058 China; 4 Department of Chemistry and Chemical Biology, Northeastern University, Boston, MA 02115 USA; 5 Institute for Auditory Neuroscience, University Medical Center Göttingen, 37077 Göttingen, Germany; 6 Leiden University Medical Center, Einthovenweg 20, 2333 Leiden, ZC the Netherlands; 7 Department of Systems Biology, Harvard Medical School, Boston, MA 02115 USA; 8 Department of New Biology, Daegu Gyeongbuk Institute of Science and Technology (DGIST), Daegu, 42988 Korea; 9 Biospectroscopy, Center for Protein Diagnostics (PRODI), Ruhr University Bochum, 44801 Bochum, Germany; 10 Department of Biophysics, Ruhr University Bochum, 44801 Bochum, Germany; 11 Multiscale Bioimaging: from Molecular Machines to Networks of Excitable Cells (MBExC), University of Goettingen, 37073 Göttingen, Germany

**Keywords:** Deubiquitylating enzymes, Proteasome, Cryoelectron microscopy

## Abstract

The proteasome recognizes ubiquitinated proteins and can also edit ubiquitin marks, allowing substrates to be rejected based on ubiquitin chain topology. In yeast, editing is mediated by deubiquitinating enzyme Ubp6. The proteasome activates Ubp6, whereas Ubp6 inhibits the proteasome through deubiquitination and a noncatalytic effect. Here, we report cryo-EM structures of the proteasome bound to Ubp6, based on which we identify mutants in Ubp6 and proteasome subunit Rpt1 that abrogate Ubp6 activation. The Ubp6 mutations define a conserved region that we term the ILR element. The ILR is found within the BL1 loop, which obstructs the catalytic groove in free Ubp6. Rpt1-ILR interaction opens the groove by rearranging not only BL1 but also a previously undescribed network of three interconnected active-site-blocking loops. Ubp6 activation and noncatalytic proteasome inhibition are linked in that they are eliminated by the same mutations. Ubp6 and ubiquitin together drive proteasomes into a unique conformation associated with proteasome inhibition. Thus, a multicomponent allosteric switch exerts simultaneous control over both Ubp6 and the proteasome.

## Abstract (web summary)

The interplay of the proteasome and deubiquitinase Ubp6 is crucial for the degradation of ubiquitylated substrates. Here, the authors provide structural insights into the allosteric mechanism by which the activities of both Ubp6 and the proteasome are regulated.

## Introduction

The proteasome is the most complex protease known, the primary ubiquitin-dependent protease in eukaryotic cells, and a well-established target of anti-cancer drugs^1–3^. It plays a major role in myriad regulatory processes and stress responses. Ubiquitinated substrates are first recognized by the 19-subunit proteasome regulatory particle (RP), then translocated through a channel into the proteasome core particle (CP) to be degraded. The channel is narrow so as to restrict translocation to proteins that are unfolded or that can be actively unfolded by the proteasome^4,5^. Unfolding is driven by the ATPases Rpt1-Rpt6, which form a heteromeric ring complex within the RP, the center of this ring defining the substrate’s path to the CP. Because ubiquitin has an exceptionally stable structure^5,6^, ubiquitin modifications on a substrate constitute a kinetic impediment to translocation^7–10^. Thus, substrates are typically deubiquitinated prior to the completion of translocation. The release of ubiquitin both spares it from degradation and provides a checkpoint for the control of proteasome output^11^.

Indiscriminate deubiquitination at the proteasome would potentially lead to premature removal of ubiquitin and inefficient proteasome function. Therefore the deubiquitinating activity of the proteasome is expected to be highly controlled. In budding yeast, two deubiquitinating enzymes reside on the proteasome: Rpn11, an integral subunit of the RP, and Ubp6 (whose mammalian ortholog is USP14), a nonstoichiometric factor that binds the RP reversibly. Rpn11 is positioned directly above the substrate entry port of the proteasome^12–16^, so that ubiquitin chains bound to the substrate are inevitably brought to its active site as the substrate undergoes ATP-dependent translocation. Since Rpn11 depends on translocation, it acts primarily on substrates committed to be degraded, and promotes rather than inhibits substrate degradation^8,10,17^.

The deubiquitinating activity of Ubp6, in contrast to that of Rpn11, is not ATP-dependent or linked to substrate translocation. With a favorable in vitro substrate, ubiquitin removal by Ubp6 can be detected in less than a second, and achieved before the proteasome initiates degradation^18^. Thus, Ubp6 can suppress degradation through kinetic competition with the proteasome. Only a subset of proteasome substrates is subject to this effect, apparently because the substrate requirements of Ubp6 are stringent. Preferred in vitro substrates carry multiple ubiquitin chains, which are removed by Ubp6 *en bloc* until a single chain remains, which is then resistant to its action^18^.

In the absence of its catalytic activity, Ubp6 remains capable of suppressing protein degradation by the proteasome^19,20^. This second, noncatalytic mode of inhibition is promoted by binding of ubiquitin to its active site^19,20^. Ubiquitin “loading” of Ubp6 and USP14 also appears to promote docking of the catalytic domain of the enzyme near the exterior face of the oligosaccharide-binding (OB) domain of subunit Rpt1^19,21,22^. The functional consequences of this interaction have not been examined.

Ubp6 and USP14 are among the most tightly and intricately regulated deubiquitinating enzymes. They are activated by the proteasome, an 800-fold effect in the case of USP14^23^. These enzymes are also controlled by stresses^24,25^; by regulated recruitment to the proteasome^26–28^; by AKT-dependent phosphorylation, mediating metabolic control of USP14 activity;^29^ and by multiple microRNAs^30–34^. The intricate regulation of Ubp6 and USP14 points to their importance as general modulators of the output of the ubiquitin-proteasome system.

In this study, we identify the contact site between the proteasome and the catalytic domain of Ubp6 by mutation and show that it functions as a bidirectional switch controlling the activity of not only Ubp6 but of the proteasome as well. The switch involves two previously unknown functionalities: the ILR element of Ubp6 and the L34 activation loop of Rpt1. These mutations also target the noncatalytic activity of Ubp6, and thus point to an essential linkage between the catalytic and noncatalytic activities of Ubp6. By delaying proteasome-mediated substrate degradation, the noncatalytic effect of Ubp6 may impose temporal order on otherwise competing for enzymatic reactions, and provide an extended, substrate-controlled time window for Ubp6 to remove ubiquitin groups.

## Results

### Ubp6 mutants defective in activation

**Fig. 1:**
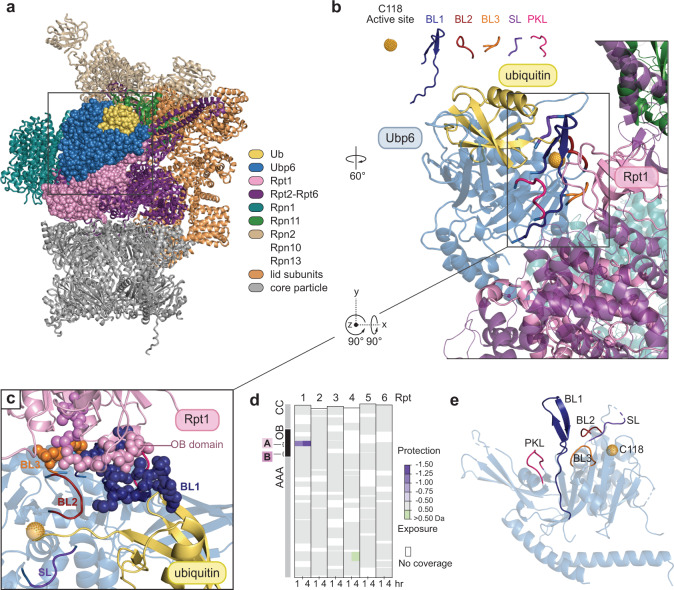
Model of the Ubp6-Rpt1 interface. **a** Model of the Ubp6-UbVS-proteasome complex based on cryo-EM analysis. Ubp6, Rpt1, and ubiquitin are highlighted as spheres, other proteins as ribbons. Rpn1, teal; Rpn11, green; Rpn10 and other base subunits, tan; lid components, light brown; ATPases other than Rpt1, purple; core particle, gray. Boxed region is rotated and enlarged in *b*. (PDB: 7QO3, 7QO4). **b** Detail of Ubp6-Rpt1 interface with key features of Ubp6 highlighted. **c** Enlarged underside view of the Ubp6-Rpt1 interface with mutagenized residues rendered as spheres. Interface A is in blue for Ubp6 and pink for Rpt1; Interface B, orange for Ubp6, violet for Rpt1. **d** Interface A is protected from hydrogen exchange by the addition of excess Ubp6-UbVME. Ubp6-dependent deuteration differences within peptides of Rpt1-Rpt6 of purified regulatory particle are shown. See Supplementary Fig. 5 for additional HDX-MS data. All deuterium uptake values used to generate these difference maps can be found in Supplementary Data 1. **e** Critical loops of free Ubp6 (PDB: 1VJV).

We performed cryo-EM analysis of the proteasome complexed to Ubp6 covalently bound through its active site cysteine to ubiquitin-vinyl-sulfone (UbVS), achieving structural insights at better resolution (6 to 7 Å) than previously reported (9.5 Å)^21^ for this complex (Fig. 1a, Supplementary Figs. 1–3, Supplementary Table 1). The improvement in resolution enabled us to distinguish the different conformational states of this complex. Details of these ternary complexes will be presented below; we will focus initially on targeted mutagenesis based on these structures. Ubp6 has two domains: an N-terminal ubiquitin-like (UBL) domain, which binds the proteasome via subunit Rpn1^35–37^, and a C-terminal catalytic domain, which contacts Rpt1 (Supplementary Fig. 4a). The Rpt1 contact site^21^ (Fig. 1b, c) comprises Interfaces A (R316-V333 of Ubp6 and G158-E169 of Rpt1) and B (E473-S488 of Ubp6 and Y181-R190 of Rpt1). We validated Interface A by showing that it is uniquely protected from hydrogen deuterium exchange within Ubp6-ubiquitin-vinyl-methyl-ester (UbVME)-RP ternary complexes (Fig. 1d, Supplementary Fig. 5).

Interface A of Ubp6 lies within blocking loop 1 (BL1), which, in the absence of the proteasome, occludes the active site groove of the enzyme^38^ (Supplementary Figs. 4a, 6a). BL1 is rearranged to interpose between ubiquitin and the proteasome in the ternary complex (Fig. 1b, c). Interface B of Ubp6 is formed by the BL3 loop (Fig. 1c, Supplementary Fig. 4a). BL3 does not occlude the active site of free Ubp6 but rather contacts BL1 at its foot, as does the PKL loop on the opposite side of BL1 (Fig. 1e). BL1 is thus immobilized in free Ubp6. For ubiquitin to access the active site, two additional loops, BL2 and switching loop (SL), must be displaced (Supplementary Fig. 6a). However, unlike BL1 these loops are not in contact with the proteasome (Fig. 1b), suggesting that BL1 may convey the proteasome’s signal for activation to these elements. We refer to BL1, BL2, and SL collectively as the blocking loops. They are all strongly conserved in evolution, indicating their functional importance (Supplementary Fig. 6b).

**Fig. 2:**
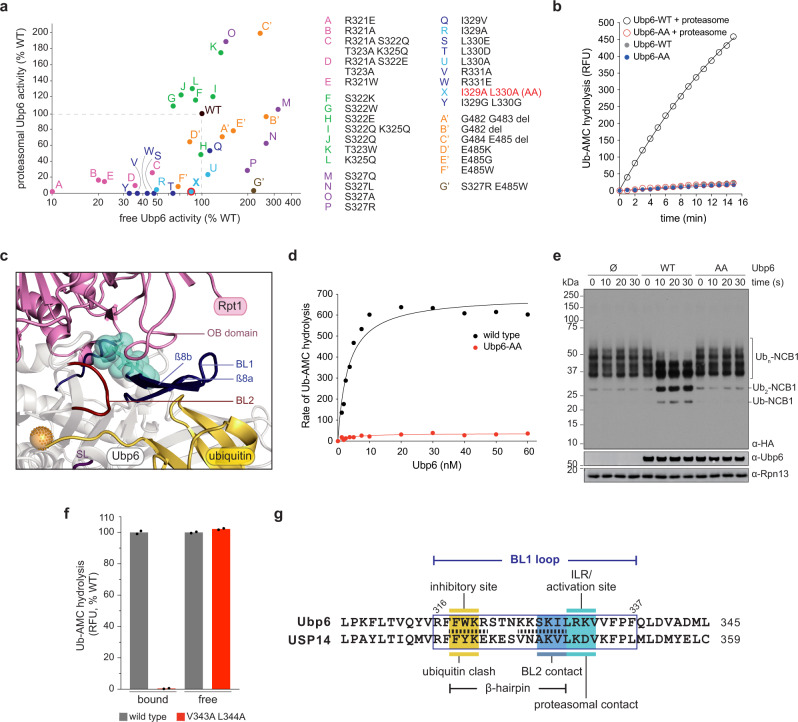
Ubp6 and USP14 mutants that are refractory to proteasome activation. **a** Activity of Ubp6 mutants in the presence or absence of proteasome, with Ubp6-AA highlighted. **b** Ub-AMC hydrolysis by Ubp6-AA (60 nM). **c** Positions of I329 and L330 (cyan) in the modeled Ubp6-Rpt1 interface. (PDB: 7QO3, 7QO4). **d** Concentration-dependence of Ubp6 activity on Ub-AMC (1 μM) in the presence of *ubp6*Δ *hul5*Δ proteasome (1 nM). The data were fit to a hyperbolic curve, yielding a K_d_ of 3.3 nM for wild-type Ubp6 and 4.0 nM for Ubp6-AA. See also Supplementary Fig. 7. **e** Activity of Ubp6-AA on HA-Ub_n_-NCB1 in the presence of proteasome (and ADP to prevent substrate degradation). Such deubiquitination is strictly proteasome dependent^18^. **f** Activity of the USP14-V343A L344A with or without proteasome. Wild-type values are independently set to 100% but differ by over 100-fold, reflecting the extent of activation. *n* = 2 independent samples per experiment were examined, normalized mean activity is plotted. This experiment was repeated independently with closely consistent results. **g** Sequence alignment between Ubp6 and USP14. Ubiquitin clash for free Ubp6 is shown in Supplementary Fig. 6a. Stippled lines, β-strand-forming residues in Ubp6 (see Supplementary Fig. 6c). Figure 5e describes BL2 contacts. Source data are provided as a Source Data file.

Interfaces A and B were chosen for mutagenesis (Fig. 2a). We screened for mutants in which the activity of proteasome bound Ubp6 is reduced while that of free Ubp6 is minimally affected (Fig. 2a, Supplementary Fig. 7a, b). The *ubp6-I329A L330A* mutant (hereafter *ubp6-AA*) exhibited nearly ideal behavior (Fig. 2b), with a stringent reduction of proteasome-activated deubiquitinating activity (to ~2% of WT), and preservation of free activity. I329 and L330 are in close contact with Rpt1 in the structural model (Fig. 2c). Loss of deubiquitinating activity in the mutant could not be corrected by increasing the concentration of Ubp6; only a slight reduction of the affinity of Ubp6 for the proteasome was apparent (Fig. 2d and Supplementary Fig. 7c). Since Ub-AMC is not a true ubiquitin-protein conjugate, we tested Ubp6-AA on a ubiquitinated fragment of cyclin B^18^ (Ub_n_-NCB1). No deubiquitination was detected in the presence of Ubp6-AA (Fig. 2e). We also validated the *ubp6-AA* mutant by showing that it phenocopies *ubp6*Δ in vivo (Supplementary Fig. 7d, e).

To test whether the activation mechanism of Ubp6 is evolutionarily conserved, we generated substitutions in USP14 (human form), targeting the cognate residues of I329 and L330. Proteasome dependent activation was reduced in the USP14-V343A L344A mutant to ~0.4% of wild-type, while the basal activity of free USP14 was unaffected (Fig. 2f and Supplementary Fig. 7f, g). Thus, the mutations identify a conserved mediator of Ubp6 activation, which we term the ILR element (Fig. 2g).

### Elements of the BL1 loop

Although the ILR element is within the BL1 loop, it is ten residues removed from the segment of BL1 that occludes ubiquitin access to the active site groove (Fig. 2g). How does the ILR reposition the inhibitory site of BL1? Fig. 2c shows that the BL1 loop forms a previously unrecognized ß-hairpin (see Supplementary Fig. 6c for structural details). Ubiquitin is occluded by the ß8a strand of the hairpin; therefore, activation should involve directed movement of this strand. The connection of ß8a to I329 and L330 is provided by ß8b, which is directly abutted by I329 and L330. Thus, we propose that the BL1 loop contains three distinct elements–the ILR, ß8a, and ß8b–which function in cooperation to control of Ubp6 activity (Fig. 2g). The sister strands of the BL1 loop are densely interconnected, so that the loop functions as a relatively rigid lever arm (Supplementary Fig. 6c, d) that efficiently propagates the allosteric signal emanating from the ILR element.

Repositioning of ß8a is expected to be insufficient to allow ubiquitin docking, as ubiquitin occlusion by the BL2 and SL would also have to be relieved (Supplementary Fig. 6a). However, unlike BL1, BL2 and SL do not contact the proteasome in our model (Fig. 1b). Examination of the crystal structure of free Ubp6 revealed that BL1 and BL2 are in direct contact, and SL is in contact with BL2, suggesting that transition of BL1 to the open form promotes the same change of state for the other blocking loops, as discussed below.

### Rpt1 mutant defective in Ubp6 activation

**Fig. 3:**
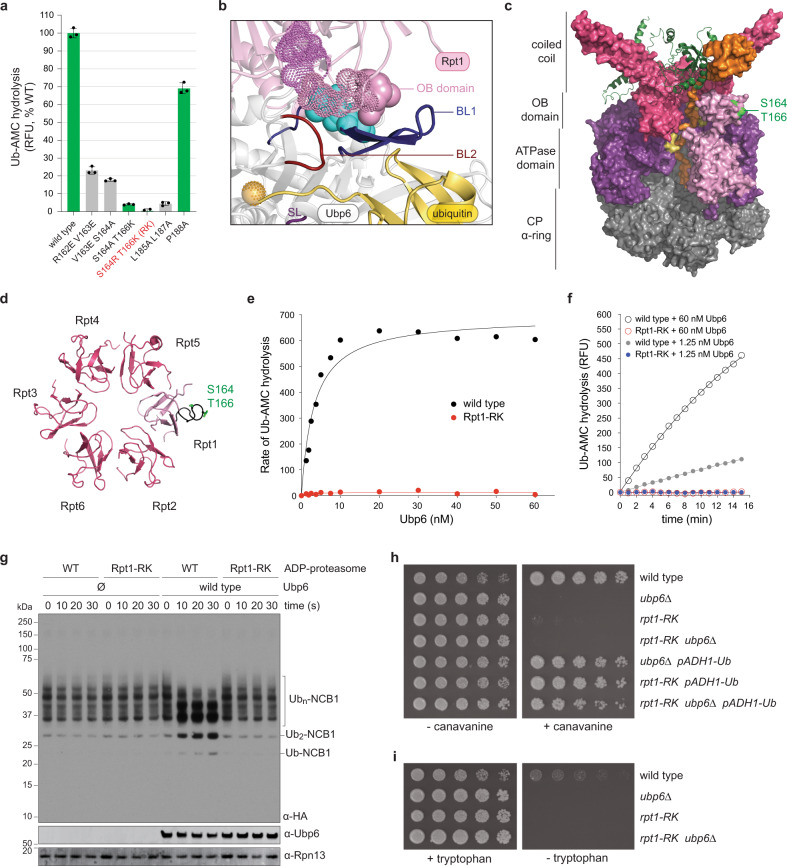
An Rpt1 mutant defective in Ubp6 activation. **a** Ub-AMC hydrolysis by Ubp6 in the presence of wild-type and mutant proteasomes. *n* = 3 independent samples per experiment were examined. Data are presented as mean values ± SD. *rpt1-RK* is highlighted. Mutants in green showed unperturbed proteasome assembly (see Supplementary Fig. 8); the assembly was impaired for those in gray. **b** The modeled Rpt1-Ubp6 interface. Cyan, Ubp6 residues I329 and L330. Solid pink, Rpt1 S164 and T166. Stippled pink and violet spheres, other mutated Rpt1 residues (PDB: 7QO3, 7QO4). **c** Positions of S164 and T166 (neon green) within the OB domain of the ATPase ring. Coiled-coil and OB domains are in rose, ATPase domain in purple, Rpt1 in pink, Rpn11 in green ribbon. Substrate (orange) is passing through RPT pore loops (yellow) into the CP axial channel. Portions of Rpt1 and Rpt2 were removed to reveal the substrate channel. (PDB: 6EF3). **d** Top view of the OB domain. Rpt1’s L34 loop is in black, residues S164 and T166 in green. (PDB: 6EF3). **e** The effect of Ubp6 concentration on Ub-AMC hydrolysis in the presence of proteasome (1 nM) purified from *ubp6*Δ *hul5*Δ (nominally wild-type) or *rpt1-RK* *ubp6*Δ *hul5*Δ strain. Data were fit to a hyperbolic curve, yielding a K_d_ value of ~3.3 nM and ~2.0 nM for wild-type and Rpt1-RK proteasome, respectively. **f** Ubp6 activity in the presence of mutant proteasomes. **g** Deubiquitination of HA-Ub_n_-NCB1 in the presence of Ubp6 and ADP-proteasomes. **h** Wild-type, *ubp6*Δ, and *rpt1-RK* mutants were serially diluted and spotted onto agar plates in the presence or absence of canavanine (1.5 μg/mL). The pADH1-Ub transgene is integrated into the *UBP6* locus and expresses ubiquitin from the *ADH1* promoter. **i** Ub-K-Trp reporter stabilization by *rpt1-RK* mutant. Yeast cells were serially diluted and plated on media containing or lacking tryptophan. Source data are provided as a Source Data File.

The Rpt1 components of Interfaces A and B lie within its OB domain (Fig. 1c, d), one of six proteasomal OB domains, which form a ring complex defining the substrate entry port of the RP^39^, but is to date not known to have any catalytic or regulatory function. To identify the proteasomal receptor site of the Ubp6 catalytic domain, OB domain mutants covering Interfaces A and B were generated. Several mutations impaired proteasome assembly and were not further studied (Fig. 3a and Supplementary Fig. 8). Among assembly-proficient mutants, two were almost completely defective in activation of wild-type Ubp6: *rpt1-S164R T166K* and *rpt1-S164A T166K* (Fig. 3a). S164 and T166 are proximal to I329 and L330 of Ubp6 in our structural model (Fig. 3b). The *S164R T166K* double mutant (hereafter *rpt1-RK*), which has lost ~97% of its capacity to activate Ubp6, was chosen for further analysis. S164 and T166 fall within a segment of the conserved L34 loop of the Rpt1 OB domain^39^ (Fig. 3c, d), hereafter termed the activation loop.

Rpt1-RK proteasomes appeared to be inherently unable to activate Ubp6, and not simply attenuated in Ubp6-proteasome affinity, as the catalytic defect could not be overcome by adding elevated levels of Ubp6 to the reaction (Fig. 3e, f). The failure in deubiquitination extended to bona fide ubiquitin-protein conjugates (Fig. 3g) and was confirmed by in vivo assays (Fig. 3h, i).

### Relief of proteasome inhibition

**Fig. 4:**
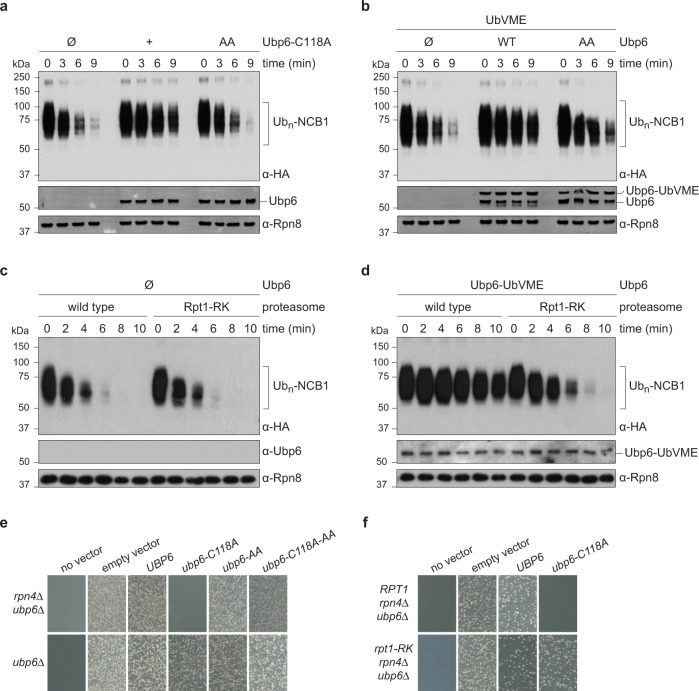
Ubp6-AA and Rpt1-RK mutants are impaired in the Ubp6 noncatalytic effect. **a** In vitro degradation assay performed with C118A or the C118A I329A L330A triple mutant of Ubp6, proteasome, and HA-Ub_n_-NCB1 (detected with anti-HA antibody). Ubp6 and Rpn8 are loading controls. **b** Inhibition of HA-Ub_n_-NCB1 degradation by Ubp6-UbVME. UbVME was preincubated with Ubp6 variants to covalently modify C118, abolishing deubiquitinating activity. The assay was otherwise as in *a*. **c** HA-Ub_n_-NCB1 degradation by wild-type and mutant proteasomes. **d** As *c* but with Ubp6-UbVME added. All in vitro assays have been independently repeated. **e** In vivo assay of the Ubp6 noncatalytic effect. Plasmids expressing variants of Ubp6 were transformed into either *ubp6*Δ *rpn4*Δ or *ubp6*Δ yeast strains, and colony formation was recorded after incubation at 30 °C for 3–4 days. Loss of noncatalytic activity was seen in *ubp6-C118A-AA*. **f** The noncatalytic effect of *ubp6-C118A* is abrogated by the *rpt1-RK* mutation.

When the catalytic cysteine of Ubp6 is substituted with alanine (Ubp6-C118A), the resulting enzymatically inactive protein still inhibits the proteasome^20^. This “noncatalytic effect” is not an aberrant feature of the mutant, but an inherent property of Ubp6, since it is also seen with wild-type Ubp6 when it is coupled to ubiquitin in adducts such as Ubp6-UbVS^19,20^. Are mutants in which the proteasome cannot activate Ubp6 also defective in proteasome inhibition? To test this possibility, in vitro degradation assays were performed using Ub_n_-NCB1 as substrate. We observed a strong noncatalytic effect when degradation assays were performed in the presence of Ubp6-C118A (Fig. 4a). The effect was almost completely abrogated by the Ubp6-C118A-AA triple mutant protein. Failure of the noncatalytic effect could simply result from deficient ubiquitin engagement by this mutant. However, when we used Ubp6-AA, modified covalently at Cys118 by UbVME, we similarly observed a strong impairment of the noncatalytic effect (Fig. 4b). Thus, even when ubiquitin occupies the active site of Ubp6, forcing the blocking loops open, the Ubp6-AA mutant protein cannot inhibit the proteasome.

The Rpt1-RK mutant proteasome was indistinguishable from wild-type when tested in a degradation assay with Ub_n_-NCB1 as substrate and no Ubp6 present (Fig. 4c), exemplifying the specific nature of this mutant. However, the mutant restored substrate degradation in the presence of Ubp6 (Fig. 4d). Thus, the L34 activation loop serves as the receptor element in the proteasome for the noncatalytic effect exerted by Ubp6. In both activation of Ubp6-mediated deubiquitination and the noncatalytic effect, the *rpt1-RK* mutant phenocopies *ubp6-AA*. Abrogation of the noncatalytic effect was also shown in vivo for both mutants (Fig. 4e, f). In summary, these results indicate that Ubp6 activation and proteasomal inhibition are inherently coupled processes.

### Structure of Ubp6-inhibited proteasomes

**Fig. 5:**
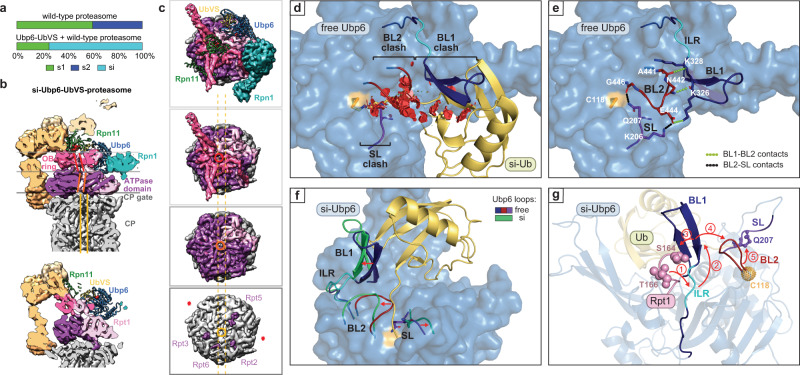
The inhibitory Ubp6-UbVS-proteasome complex defines a new conformational state of the proteasome. **a** Ubp6-UbVS induces the si proteasome state. **b** Cross-section of the cryo-EM density map of the si state proteasome at 7.0 Å resolution for the ATPase domains including Ubp6 and 6.1 Å for the rest. Misaligned axial channels of the OB ring, the ATPase domain ring, and the CP are denoted by parallel bars (red, orange, and yellow, respectively). Rpn11 (green) is misaligned with the OB domain substrate entry port. Horizontal lines indicate cutting planes in *c*. Bottom: alternative z-plane visualizes Ubp6 catalytic domain. Active sites of Ubp6 and Rpn11 are highlighted in red, proteasome subunits as in Fig. 1a. **c** Cut-away views down the long axis of si proteasome from the OB ring to the ATPase domain ring to the CP α-ring. Subunits of interest are highlighted. RP axial channels are off-axis to the CP (dotted yellow lines). The OB substrate entry port is circled in red, ATPase translocation channel in orange. Four C-terminal Rpt tails (Rpt2, Rpt6, Rpt3, Rpt5) are inserted into CP α pockets. Asterisks: unoccupied α pockets. **d** Occlusion of ubiquitin by BL1, BL2, and SL in free Ubp6 illustrated by red disks (PDB: 1VJV). Ubiquitin is modeled onto free Ubp6, positioned as in complex si. **e** BL1-BL2-SL network in free Ubp6 (PDB: 1VJV) is established through interloop contacts. **f** Comparison of the blocking loop network in free Ubp6 (PDB: 1VJV) and complex si. Loops from free Ubp6 are superimposed onto si-Ubp6. The structure is rotated 90° counterclockwise from *d*. **g** Proposed cascade of signal transfer within Ubp6 upon interaction with proteasome.

After 3D classification of the proteasome-Ubp6-UbVS ternary complex dataset, we obtained two conformational states, s1 and a previously undescribed conformational state that we term si. More than 70% of the proteasomes exhibit the si structure. By applying a 2-body refinement separating the Ubp6, Ub, Rpn1, and ATPases density (body2) from the rest of the single-capped proteasome (body1), reaching resolution at 7.0 and 6.1 Å, respectively. In si state proteasomes, the catalytic domain of Ubp6 docked at Rpt1 and exerted a dramatic influence on the structure of the proteasome (Fig. 5a, b). Proteasomes in the basal state, s1, exhibit axial misalignment–a signature of their inactivity. This is also true of proteasomes in the s2 and s5 states^40–42^. With substrate engagement and conversion to an active state such as s3 or s4, co-axial positions are assumed by the active site of Rpn11, the substrate entry port of the OB ring, the central channel of the ring formed by the six ATPase domains, and the heptameric α ring of the CP^7,43^. These structural elements are misaligned in si proteasomes (Fig. 5b, c). Also indicative of an inactive state is the closed gate of the CP (Fig. 5c). The positioning of the lid of si resembles that of s5^40^, whereas the ATPase ring is comparable to that of s2, except that the C-terminal tail of the Rpt6 is inserted into the α2/α3 pocket of the CP α ring, as seen in s3 (Supplementary Fig. 9). In summary, si proteasomes borrow features from a variety of other states to form a unique and degradation-inhibited conformational state.

In free Ubp6, BL1 is stabilized at its base on opposite sides by the PKL and BL3 loops, which notably both contact the ILR element (Supplementary Fig. 6e). Although our resolution was limited, we observed that the free Ubp6 structure does not fit into the EM density and that the covalently linked ubiquitin density clashes with the BL loops (Supplementary Figs. 10, 11). The PKL and BL3 loops move away from the ILR element in the si complex, which may facilitate contact with the activation loop of Rpt1 and movement of the ILR element (Supplementary Fig. 6f).

Cryo-EM analysis of the Rpt1-RK proteasome together with wild-type Ubp6 and UbVS revealed that the fraction of proteasomes in the si state was reduced to ~25%, whereas the conformational profile of the Rpt1-RK proteasome alone was comparable to that of wild-type proteasomes (Supplementary Fig. 12a)^41^. Thus, the Rpt1 mutation impairs the Ubp6-dependent transition of the proteasome to the si state, although Ubp6-UbVS remains docked at Rpt1 in the s2 state proteasomes that are observed with the mutant (Supplementary Figs. 3e, 9d). Release of the proteasome from the si state may account for the recovery of protein degradation by the mutant (Fig. 4). The mutation also conferred structural changes on the associated Ubp6 enzyme: Ubp6 was slightly shifted from its position on wild-type proteasomes, and the activation loop retracted from the ILR (Supplementary Fig. 12b–d). In summary, a modest perturbation of Rpt1-ILR contact interface can decisively alter the conformational profile of the proteasome as a whole.

### The suppressive network of Ubp6

Comparison of the structure of Ubp6 associated with si proteasomes to the 1.7 Å structure of free Ubp6 provided major insights into the mechanism of Ubp6 activation. BL1, BL2, and SL are all in position to clash with ubiquitin density in free Ubp6 and must be displaced to activate the enzyme (Fig. 5d, Supplementary Fig. 10). In free Ubp6, BL1 directly contacts BL2 through three hydrogen bonds, extending from ß8b, directly adjacent to the ILR element (Fig. 5e). BL2, in turn, contacts SL through a salt bridge; while G446, immediately flanking BL2, directly contacts the side chain of Q207, which is the key ubiquitin-blocking residue of SL (Fig. 5d, e and Supplementary Fig. 6a). Thus, BL1, BL2, and SL stabilize each other to form an inhibitory network of blocking elements, accounting for the tight suppression of activity in free Ubp6. In the activated Ubp6 of the si structure, all three blocking loops are withdrawn from the catalytic groove (Fig. 5f, Supplementary Fig. 11). Proteasome contact with the ILR may direct repositioning of the ß-hairpin, with movements propagated in an ordered sequence across the network of blocking loops (Fig. 5g). The direct target of ILR movement is ß8b, opposite sides of which are in contact with ubiquitin-blocking elements ß8a and BL2.

## Discussion

We have identified an allosteric network composed of the activation loop of Rpt1; its target, the ILR element of Ubp6; and downstream elements, the BL1 ß-hairpin, the BL2 loop, and the SL loop. We propose that the signal generated by Rpt1-Ubp6 interaction is propagated stepwise across these elements, in a progression initiating at Rpt1 and ending at the SL loop. Network components exert control over both Ubp6 and the proteasome, activating the former and inhibiting the latter. This coupling is associated with the si state of the proteasome, which is induced by the ILR allosteric switch. The pausing of proteasome-mediated substrate degradation in the si state may impose temporal order on otherwise competing for enzymatic reactions, and provide an extended, substrate-controlled time window for Ubp6 to remove ubiquitin groups. While the key features of this allosteric switch are clear from the properties of the mutants reported here and from our structural analysis, certain details underlying this switch remain to be understood. A clear-cut validation of the precise side chain interactions within the Ubp6 proteasomal complex will require structural information of the complex at least below 3 Å resolution.

**Fig. 6:**
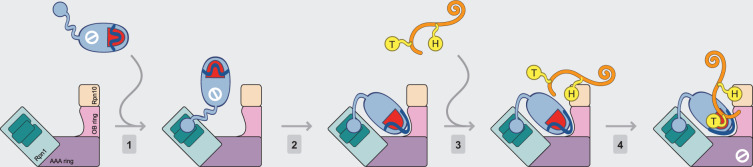
Model for assembly of the Ubp6 catalytic complex. Free Ubp6 has an inactive conformation (slashed circle), its active site (red semioval) being blocked by BL1, BL2, and SL (blue wave). Step 1, Ubp6 and the proteasome are complexed via the Ubp6^UBL^-Rpn1 interaction^2^. For clarity, only a fraction of proteasome subunits are represented. The Ubp6^UBL^-Rpn1 interaction does not activate Ubp6 (ref. ^35^) but is proposed to promote association of the Ubp6 catalytic domain to Rpt1 through avidity (step 2). Ubp6-Rpt1 interaction in this context may partially destabilize the blocking loop network to enable ubiquitin loading; this remains conjectural. Activated Ubp6 remains highly selective in that it will efficiently cleave only ubiquitin-protein conjugates that carry more than one ubiquitin modification^18^. Thus, a second docking event wherein substrate-bound “helper ubiquitin” (H) is docked at a ubiquitin receptor such as Rpn10, is required (step 3). With step 4, docking of the “target ubiquitin” (T), the catalytic complex is assembled: blocking loops are displaced more completely, and the proteasome assumes the si state, imposing noncatalytic proteasome inhibition. Thus, assembly of a competent catalytic complex essentially requires three docking events involving ubiquitin or ubiquitin-like protein domains: Ubp6^UBL^ at Rpn1, helper ubiquitin at a ubiquitin receptor, and target ubiquitin in the Ubp6 active site.

Our model for Ubp6 activation on the proteasome, shown in Fig. 6, incorporates previous findings and highlights the varied roles played by ubiquitin and ubiquitin-like (UBL) domains in the process. In the absence of its N-terminal UBL domain, Ubp6 exhibits only basal activity in vitro, and the UBL deletion behaves as a null mutation in vivo^35^. The UBL docks at the T2 site of Rpn1, mutation of which also phenocopies a *ubp6* null^36^. Thus, assembly of the catalytic complex is proposed to begin with the UBL docking at T2 (Fig. 6, step 1). The interaction between Ubp6 and Rpn1 appears to promote Ubp6-Rpt1 interaction by increasing the local concentration of the Ubp6 catalytic domain as well as orienting the domain toward Rpt1.

The following steps entail displacement of the blocking loops of Ubp6 (Fig. 6). In this model, the loops are first partially displaced in a hypothetical priming reaction that will be discussed more fully elsewhere. Partial blocking loop displacement enables target ubiquitin (T) to dock at the Ubp6 active site. In step 3, the proteolytic substrate is docked on the proteasome, though not to Ubp6; initial docking involves a second ubiquitin on the conjugate (helper ubiquitin, H), which cannot be part of the same ubiquitin chain as target ubiquitin^18^. Helper ubiquitin is proposed to bind a ubiquitin receptor on the proteasome, thus driving complex assembly through avidity, similarly to the UBL domain of Ubp6. Thus, even in the primed complex, productive docking of target ubiquitin to Ubp6 remains highly constrained by the requirement for helper ubiquitin, and will not take place for many proteasome substrates. Helper ubiquitin has been shown to be important for both Ubp6 and USP14, but whether it is required for all substrates remains to be established.

After docking of target ubiquitin (Fig. 6, step 4), the final complex has the UBL of Ubp6 on Rpn1, target ubiquitin on Ubp6, and helper ubiquitin (or ubiquitin chain) engaged with a ubiquitin receptor. With the completion of this step, the proteasome assumes the si state and substrate degradation is suspended until deubiquitination takes place and target ubiquitin is released from the active site of Ubp6. After this release, the proteasome substrate has alternative fates; its degradation by the proteasome may proceed, or it may dissociate from the proteasome. The partitioning between these fates will likely depend on the number and arrangement of remaining ubiquitin groups on the proteasome substrate^18^. If, after deubiquitination, the substrate still carries multiple ubiquitin modifications, it may be subjected to successive rounds of deubiquitination by Ubp6.

Noncatalytic inhibition of the proteasome by Ubp6 is coupled to deubiquitination in that it is associated with the si state of the proteasome, in which ubiquitin is docked at the Ubp6 active site. The highly specific interactions that underlie proteasome inhibition by Ubp6 are exemplified by the ILR mutant Ubp6-AA and the Rpt1 activation loop mutant Rpt1-RK, both of which dramatically impair the noncatalytic effect even when ubiquitin has been chemically engineered to dock into the Ubp6 active site. Ubiquitin would normally not load onto Ubp6 in these mutants because they are defective in Ubp6 activation. The control of proteasome conformation by Ubp6 is expected to coordinate Ubp6 activity with other substrate processing events carried out by the proteasome. This mechanism grants time for Ubp6 to catalyze deubiquitination prior to substrate degradation, which should enhance the efficiency of chain removal by Ubp6.

Our findings may have general implications for the regulation of deubiquitinating enzymes of the 56-member USP family^44^. Since promiscuous deubiquitination by these enzymes has the potential to neutralize the myriad functions of ubiquitination, it is essential that their activities are held under negative control and allosterically activated with specificity at a given time or location. Crystallographic studies from the Shi lab^38,45^ identified blocking loops in these enzymes, BL1 and BL2, and hypothesized that they may be involved in control of activity. This idea remained hypothetical, and, surprisingly, subsequent studies have instead favored the view that blocking of the catalytic cleft by BL1 and BL2 is not critical for suppression per se. In this interpretation, BL1 and BL2 are found in an open state in substrate-engaged forms of USP enzymes, such as UbVS-modified USPs, simply by virtue of substrate accommodation^46–49^. However, a more notable feature of many deubiquitinating enzymes of the USP family is their control through allostery. Moreover, the blocking loops are primordial features of these enzymes, and are conserved over the eukaryotic kingdom and the USP family as a whole. Thus, our finding that these loops are central to allosteric control in Ubp6 suggests an important paradigm for this enzyme family.

Our findings indicate that BL1, BL2, and SL operate as an integrated network, with BL1 serving as the fulcrum for rearrangement of BL2 and SL. The participation of three distinct loops in blocking the catalytic cleft may ensure tight negative control. In USP enzymes where the evolutionary pressure to repress basal activity is less strong, some of the loops may have degenerated to a more flexible and less repressive state. Thus, while Ubp6 may provide a clear-cut case of blocking loop action because of the strength of the allosteric control mechanism, owing to the highly defined closed state of its blocking loops, the essential features of the mechanism may apply to other enzymes of the USP family. For USP14 in particular, recent work shows that the principal features of allosteric controls are comparable to those of Ubp6^50^. 

## Methods

### Expression and purification of recombinant proteins

Unless otherwise noted, Rosetta (DE3) cells (EMD Millipore) transformed with expression plasmids were grown to an OD_600_ of 0.6–0.8 in selective 2X YTG media (10 g/L yeast extract, 16 g/L tryptone, 20 g/L dextrose). Expression was induced with 0.5 mM IPTG (Gold Biotech), and cells were transferred to a 16 °C shaker for overnight induction. Cells were harvested by centrifugation, and purification was carried out as described below. All lysis buffers used were supplemented with 1X protease inhibitor cocktail^36^ and 2 mM AEBSF (Gold Biotech). A list of constructs used can be found in Supplementary Table 2. All plasmids containing Ubp6 variants were generated through conventional site-directed mutagenesis, where mutations were incorporated into amplified plasmid amplicons using phosphorylated primers carrying the desired mutation. Ligated amplicons were transformed into NEB 5-α competent cells (NEB), which were then spread onto LB agar plates carrying appropriate antibiotics for selection. Proper clones carrying targeted mutations were determined by Sanger sequencing.

#### His6-tagged Ubp6 proteins

Cell pellets were resuspended with His-tag lysis buffer (50 mM NaH_2_PO_4_, 100 mM NaCl, 10% glycerol [v/v], 25 mM imidazole [pH 8.0]). Cells were lysed by French press at 10,000 psi (two passes), and the lysate was clarified by centrifugation at 20,000 × *g* for 30 min at 4 °C. 2 mL Ni-NTA resin (Qiagen) was used for clarified cell lysate from 1 L bacterial culture. The resin was incubated with clarified lysate for 2 h at 4 °C, followed by washing with 80 bed vol of wash buffer (50 mM NaH_2_PO_4_, 300 mM NaCl, 10% glycerol [v/v], 25 mM imidazole, [pH 8.0]). Stepwise elution was achieved using a total of 10 mL elution buffer (50 mM NaH_2_PO_4_, 100 mM NaCl, 10% glycerol [v/v], 250 mM imidazole [pH 8.0]). Peak fractions containing significant amounts of protein were pooled. All Ubp6 variants were prepared using His6-tagged constructs.

#### Generation of Ubp6-UbVS and Ubp6-UbVME adduct

A 5-fold molar excess of ubiquitin-vinyl-sulfone (UbVS) or ubiquitin-vinyl-methyl-ester (UbVME) was incubated with different Ubp6 variants in reaction buffer (50 mM Tris-HCl [pH 7.5], 1 mM DTT) for 2.5 h at 30 °C. Adducts were purified by FPLC on a Superdex 200 HiLoad 16/600 column (GE Healthcare) to remove unmodified Ubp6. Purified adducts were stored in a buffer of 20 mM Tris-HCl (pH 7.5), 50 mM NaCl at −80 °C.

#### GST-tagged USP14

Pelleted cells were resuspended and lysed in PBS (137 mM NaCl, 2.7 mM KCl, 10 mM Na_2_HPO_4_, 1.8 mM KH_2_PO_4_ [pH 7.4]). 500 μL Glutathione Sepharose 4B resin (GE Healthcare) was used for clarified cell lysate from 1 L bacterial culture. The resin was incubated with clarified lysate for 2 h at 4 °C, followed by washing with 50 bed vol of PBS, then 50 bed vol of PBS with 100 mM NaCl, and lastly with 50 bed vol of PBS. To remove the GST tag, resin was incubated with 2 bed vol of PBS containing 10 μL of 1 U/μL thrombin (Sigma) for 2 h at 25 °C with occasional agitation. To remove thrombin, 100 μL Benzamidine-Sepharose (GE Healthcare) was then added, and the eluate was incubated for 30 min at 4 °C with rocking. Glycerol was added to the eluates at 10% (v/v) final concentration for storage at −80 °C.

#### Recombinant proteasome base for HDX-MS experiments

The three plasmids used for recombinant expression of yeast base subcomplex were a kind gift from Dr. A. Martin (UC Berkeley): pCOLADuet-Rpt1-Flag, His6-Rpt3, Rpt2, Rpt4-6 (kanamycin), pETDuet-Rpn1,2,13 (ampicillin) and pACYCDuet-Nas2, Nas6, Hsm3, Rpn14 (chloramphenicol), and tandem affinity purification was carried out as described^51^.

### Purification of yeast and human proteasomes

#### Purification of 26S yeast holoenzyme and proteasome subcomplexes

Protein A-tagged 26S proteasome and RP used for biochemical assays were affinity-purified as described^52^, and yeast strains used are listed in Supplementary Table 3. Purification of wild-type and Rpt1-RK mutant 26S proteasomes with a 3X FLAG tag used for cryo-electron microscopy studies was performed as described^41^, and yeast strains used for this purpose are listed in Supplementary Table 4.

#### Purification of biotin-tagged human proteasome

Human proteasome holoenzyme was purified via affinity tag as previously described^18^. HEK293T cells stably expressing epitope-tagged RPN11 (a generous gift from Dr. L. Huang^53^) were lysed by 20 strokes with a Dounce homogenizer in lysis buffer (50 mM NaH_2_PO_4_ [pH 7.5], 100 mM NaCl, 10% glycerol [v/v], 5 mM MgCl_2_, 0.5% NP-40 [v/v], 5 mM ATP, 1 mM DTT) containing protease inhibitor cocktail tablet (Roche). The total cell lysate was cleared by centrifugation at 16,000 × *g* for 15 min at 4 °C. The cleared lysates were incubated with NeutrAvidin resin (Thermo Fisher, 25 μL resin per 100 mm dish) for at least 2 h at 4 °C. The resin was extensively washed with 20 bed vol of lysis buffer, which fully removes endogenous USP14 associated with the proteasome, followed by 20 bed vol of low-salt buffer (50 mM Tris-HCl [pH 7.5], 1 mM MgCl_2_, 1 mM ATP, 10% glycerol [v/v]). Human proteasomes were eluted from the beads by cleavage, using 2 μL of 10 U/μL AcTEV protease (Thermo Fisher) in 2 bed vol of low-salt buffer supplemented with 1 mM DTT for 1 h at 30 °C. The yield from two 100 mm dishes was ~8 μg of proteasome.

To eliminate UCH-L5 activity, human proteasomes were treated with ubiquitin-vinyl-sulfone (UbVS, Boston Biochem). UbVS was added to the resin at 1–1.5 mM, followed by incubation at 30 °C for 2 h prior to TEV cleavage. Residual UbVS was removed by washing the resin with at least 20 bed vol of low-salt buffer. The Ub-AMC hydrolysis assay was used to confirm the elimination of UCH-L5 activity. The purity and integrity of the purified proteasomes were routinely assessed using native gels^54^ and SDS-PAGE.

### Cryo-electron microscopy (cryo-EM) studies

#### Sample preparation and data collection

To study the structure of the 26S-Ubp6-UbVS complex, 26S proteasomes and Ubp6-UbVS were mixed in a 1:4 ratio and incubated for 20 min in a buffer containing 20 mM HEPES-NaOH (pH 7.4), 40 mM NaCl, 4 mM DTT, 4 mM MgCl_2_, 4 mM ATP, and ~25% sucrose on ice before plunging. Cryo-EM data of the plunged samples, 26S-Ubp6-UbVS (400 nM 26S, 1.6 μM Ubp6-UbVS), free 26S Rpt1-RK (400 nM), 26S Rpt1-RK-Ubp6-UbVS (400 nM 26S Rpt1-RK, 1.6 μM Ubp6-UbVS) were collected on a Titan Krios (Thermo Fisher) with a K2 or K3 detector (Gatan Inc.). Automated data acquisition was performed using either SerialEM^55^ or Latitude S (Gatan Inc.). Images were acquired in counting mode at a pixel size of 1.09 Å for K3 camera and 1.38 Å for K2 camera (Supplementary Table 1). Each total exposure of 60 electrons per Å^2^ was fractionated into 30 frames for K3 camera, while 35 electrons per Å^2^ into 33 frames for K2 camera. Defocus ranged from −1.0 to −2.5 µm (K3) and −1.8 to 3.0 µm (K2).

#### Data processing

Initial motion correction was done by MotionCor^56^ as implemented by RELION 3.0^57^. Contrast transfer function estimation was performed by CTFFIND4^58^. Particles were picked either by REION or Cryolo^59^. The following processing was done in RELION 3.0, unless otherwise specified. Two rounds of 2D classification and one round of 3D classification were performed to enable selection of double-capped particles for further processing. A published map (EMD-3534) was low-pass filtered to 60 Å and used as a reference for initial 3D classification. A C2 symmetry expansion was performed, followed by subtraction of a single 19 S cap density from the double-capped particles. A refinement applying C2 symmetry and Bayesian polishing improved the resolution of the maps. Final classes were compared with known conformations and assigned to the conformational states. In the 26S-Ubp6-UbVS dataset, particles were distributed into two classes, s1 and a previously unassigned conformation of the 26S proteasome, which we designate si (Fig. 5). The si state is the more abundant, accounting for nearly 75% of the distribution. Particle distribution in the free 26S Rpt1-RK dataset was essentially identical to that of the free WT 26S dataset^41^. In the 26S Rpt1-RK-Ubp6-UbVS dataset, particles were distributed into s1, s2 and si (Supplementary Fig. 12). We further processed the si state of the 26S -Ubp6-UbVS sample and s2 and si states of the 26S Rpt1-RK-Ubp6-UbVS sample (Supplementary Figs. 1–3). To improve the resolution around Ubp6 in the si^WT^ structure, a 2-body refinement was performed with the si^WT^ structure, separating the flexible Rpn1, Ubp6, ubiquitin and ATPases density (body2) from the rest of the single-capped proteasome (body1). All maps were sharpened by phenix.auto sharpen^60^. Data collection and processing parameters are given in Supplementary Table 1.

### Model construction of Ubp6 bound proteasome states in cryo-EM analysis

#### Initial Ubp6 model with bound ubiquitin-vinyl-sulfone

We first constructed a complete model of the catalytic (CAT) domain of Ubp6 (residues 104 to 499) based on the 1.74 Å resolution yeast Ubp6 crystal structure with the PDB: 1VJV. Structural elements not resolved in the crystal structure (residues 200 to 203, 174 to 177, 283 to 293, and 370 to 387) were modeled using the Rosetta^61^ ab initio structure prediction framework implemented as plugin in VMD1.9.4a35 software^62^. This framework was initially developed to furnish the structurally unresolved regions of the 26S proteasome^63^. For each initially unresolved region larger than three residues, the predicted 5000 models were clustered using the partitioning around medoids cluster algorithm. The representative structure with the best score was used to complete the unresolved domains. The resulting completed model of the CAT domain of Ubp6 was then docked as rigid body into the cryo-EM density using Chimera^64^.

In all states, we identified an extra density where ubiquitin usually binds to Ubp6. So we constructed an Ubp6-CAT complex with bound ubiquitin. The human ubiquitin aldehyde bound USP14 structure (PDB: 2AYO) was aligned with the obtained Ubp6-CAT model. Then the coordinates of the ubiquitin aldehyde chain (chain ID B) of the aligned 2AYO crystal structure were pasted into our Ubp6-CAT model. The resulting Ubp6-CAT-ubiquitin-vinyl-sulfone (Ubp6-CAT-UbVS) model was then docked as rigid body into the remaining cryo-EM densities.

#### Initial 26S proteasome models

To model the CP and the lid of the 26S yeast proteasome, we used the structures of the different proteasome states without bound Ubp6 from Eisele et al.^40^ as initial structures for rigid body docking. The exact subunits used for each state are detailed in Supplementary Table 1. The Rpt1 mutants were generated using the mutagenesis plugin in VMD to replace in Rpt1 serine 164 by an arginine and threonine 166 by a lysine.

#### Model fitting into the cryo-EM maps

The aforementioned structures for each of the states were fitted into the respective cryo-EM map using molecular dynamics flexible fitting (MDFF)^40^. MDFF employs molecular dynamics to fit initial models into a density in real space, and thus permits protein flexibility while maintaining realistic protein conformations^65^. We used NAMD^66^ with the CHARMM36 force field for MDFF calculations. During MDFF runs, restraints to preserve the secondary structure, chirality, and cis-peptide bonds were applied to avoid overfitting. As further step to reduce artifacts due to overfitting, all MDFF runs were performed at a modest gscale of 0.3.

First the Ubp6-CAT models and, if available, the Ubp6-CAT-UbVS interface were fitted into the density with MDFF while fixing the rest of the 26S proteasome. We employed an additional constraint between the catalytic Cys118 of Ubp6-CAT and the C-terminus of UbVS. Such constraint is necessary, as the catalytic cysteine is trapped by UbVS in a thioester bond formed between the C-terminus of ubiquitin and Cys118, but parametrization of this type of bond is not available in the CHARMM36 force field.

In a subsequent MDFF run, the whole structure (including all 26S proteasome subunits) was refined. Each of these runs started with 200 steps of energy minimization followed by 40 ps MDFF simulation at a temperature of 300 K. The Ubp6-CAT-Rpt1 and Ubp6-CAT-UbVS interfaces were further refined using interactive MDFF to manually pull side chains to the desired regions of density, while interactively checking the cross-correlation values in VMD. Interactive MDFF runs were performed using the MDFF graphical user interface and initiated using QwikMD routines^67^.

Finally, we checked the hydrogen bond network with the reduce^68^ module of Phenix software^69^ and performed Phenix real space refinement with reference coordinate restraints (σ = 0.05) on the whole structure. We removed hydrogen atoms for model deposition in the PDB as hydrogen atoms are not resolved in any of the density maps.

#### Model reliability in the 6 to 7 Å resolution range

Overall conformational changes reflected by subunit rearrangement and backbone changes in structural elements such as the Ubp6 blocking loops are well reflected by our models and densities as shown in Supplementary Fig. 9. Side chain interactions within the structural models are mainly modeled based on the interaction parameters underlying the CHARMM36 force field and could possibly be biased based on the accuracy of the initial models. Detailed side chain interactions are usually not visible in cryo-EM maps at 6 to 7 Å resolution. To experimentally validate such detailed interactions higher resolution densities (at least below 3 Å) are necessary.

#### Structural data analysis

Structural models and density maps were analyzed and visualized using VMD, Chimera, PyMol, and Coot^70^. Interaction patterns were identified using PyContact^71^ and the contact matrix algorithm implemented in Maximoby (CHEOPS, Germany). The structures were validated using MolProbity^72^. The results of the structure validation are detailed in Supplementary Table 1.

### Hydrogen deuterium exchange mass spectrometry (HDX-MS)

#### Deuterium labeling

To monitor exchange in the RP, it was incubated alone or with a 5-fold molar excess of Ubp6-UbVME for 1 h on ice to allow for complex formation. To monitor exchange in Ubp6 and Ubp6-UbVME, His6-Ubp6 or His6-Ubp6-UbVME were incubated alone or with a 1.65-fold molar excess of proteasome base for 1 h on ice to allow for complex formation. After incubation, the complexes were diluted 12-fold with labeling buffer (10 mM HEPES-NaOD [pD 7.5] 50 mM NaCl, 50 mM KCl, 5 mM MgCl_2_, 0.5 mM EDTA, 0.5 mM ATP, 1 mM DTT, 10% glycerol [v/v], D_2_O [Cambridge Isotope Laboratories]) at 25 °C and quenched by a 2-fold dilution with ice-cold quench buffer (0.8 M guanidine hydrochloride, 0.8% formic acid [v/v], H_2_O) at time points ranging from 10 s to 4 h. Undeuterated control samples were prepared for each of the proteins alone and in complexes using the same procedure as outlined above and with buffer made using H_2_O instead of D_2_O.

#### Liquid chromatography and mass spectrometry (LC/MS)

Deuterated and undeuterated samples were digested immediately after addition of quench buffer with 10 μL of a 50% (by volume) slurry of beads coated with immobilized porcine pepsin (prepared in house) for 5 min on ice, then spin-filtered through a 0.45 μm cellulose acetate membrane at 4 °C for 30 sec at 10,000 × *g*. The flow-through was injected into an M-class Acquity UPLC with HDX technology (Waters). The cooling chamber of the UPLC system, which housed all the chromatographic elements, was held at 0.0 ± 0.1 °C for the entire time of the measurements. Peptides were trapped and desalted on a VanGuard Pre-Column trap (ACQUITY UPLC BEH C18, 1.7 μm, 2.1 × 5 mm column, Waters) for 3 min at 60 μL/min, eluted from the trap using a 5–35% gradient of acetonitrile over 18 min at a flow rate of 60 μL/min, and separated using an ACQUITY UPLC BEH C18, 1.8 µm, 1.0 × 100 mm column (Waters). The back pressure averaged ~12,650 psi at 0 °C and 5% acetonitrile : 95% water. The error of determining the deuterium levels was ± 0.15 Da in this experimental setup. A blank injection and run were performed between each sample to wash the trap column and analytical column. Mass spectra were acquired using a Waters Synapt G2-Si HDMS^E^ mass spectrometer. The mass spectrometer was calibrated with direct infusion of a solution of glu-fibrinopeptide (Sigma) at 200 fmol/μL at a flow rate of 5 μL/min prior to data collection. A conventional electrospray source was used, and the instrument scanned 0.4 scans/second over the range 50 to 2000 m/z with ion mobility enabled. The instrument configuration was the following: capillary voltage at 3.2 kV, trap collision energy at 4 V, sampling cone at 40 V, source temperature of 80 °C and desolvation temperature of 175 °C. All comparison experiments were done under identical experimental conditions such that deuterium levels were not corrected for back-exchange and are therefore reported as relative.

#### Data processing

Peptides were identified using PLGS 3.0.1 (Waters, RRID: SCR_016664, 720001408EN) using multiple replicates of undeuterated control samples. Raw MS data were imported into DynamX 3.0 (Waters, 720005145EN) and filtered as shown in Supplementary Table 5. Those peptides meeting the filtering criteria were further processed automatically by DynamX followed by manual inspection of all processing. The relative amount of deuterium in each peptide was determined by subtracting the centroid mass of the undeuterated form of each peptide from the deuterated form, at each time point, for each condition.

In addition to the descriptions above, comprehensive experimental details and parameters are provided in Supplemental Table 5, in the recommended^73^ tabular format. All HDX-MS data have been deposited to the ProteomeXchange Consortium via the PRIDE^74^ partner repository with the dataset identifier PXD029402.

### Ub-AMC hydrolysis assays

All reactions were performed in Ub-AMC assay buffer (50 mM Tris-HCl [pH 7.5], 1 mM EDTA, 1 mM ATP, 5 mM MgCl_2_, 1 mM DTT, and 1 mg/mL ovalbumin [Sigma]), with a final reaction vol of 20 μL per assay in a 384-well plate (Corning). Ub-AMC cleavage was monitored by measuring fluorescence in real time for at least 30 min at 365 nm excitation and 460 nm emission with an EnVision plate reader (Perkin Elmer, ICCB Facility, HMS). Analysis was performed using Microsoft Excel and GraphPad PRISM, the initial kinetics observed within the linear range was used for plotting. For routine assays, Ub-AMC (Boston Biochem) was used at a concentration (0.5 to 1 μM) far below the K_M_, so that activity measurements were performed under k_cat_/K_M_ conditions. All Ub-AMC assays were independently repeated and yielded consistent results.

### Ubp6 activity in mutagenesis screens

For free Ubp6 activity, Ubp6 variants were assayed at 0.5 μM in Ub-AMC assay buffer. The reaction was initiated by adding Ub-AMC to 0.5 uM. For proteasome bound Ubp6 activity, Ubp6 variants (4 nM final) were preincubated with *ubp6*Δ *hul5*Δ yeast proteasome (1 nM final) in Ub-AMC assay buffer at 25 °C for 15 min. The reaction was initiated by adding Ub-AMC to 0.5 μM.

### Measurement of Ubp6 affinity to proteasome

To estimate the affinity of Ubp6-AA for wild-type proteasome, and the affinity of wild-type Ubp6 for Rpt1-RK proteasome; the activation of Ubp6 for Ub-AMC hydrolysis was used as a proxy for the association. Assays were carried out using Ubp6 concentrations ranging from 1.25 nM to 60 nM. 1 μM Ub-AMC and 1 nM wild-type and *rpt1*-mutated *ubp6*Δ *hul5*Δ yeast proteasome were used. Hydrolysis rates of Ubp6 alone and proteasome alone were subtracted from the rates observed for Ubp6 in the presence of the proteasome. The Ubp6 concentration and rate data were fit to a hyperbolic curve by nonlinear regression using the PRISM software.

### USP14 activity assay

For free activity, USP14 variants were assayed at 1 μM in Ub-AMC assay buffer. The reaction was initiated by adding Ub-AMC to 1.5 μM. For proteasome bound activity, USP14 variants (8 nM final) were preincubated with UbVS-treated human proteasome (1 nM final) in Ub-AMC assay buffer at 25 °C for 15 min. The reaction was initiated by adding Ub-AMC to 1 μM.

### Preparation of APC/C-mediated ubiquitinated conjugates

APC/C was immunopurified from 100 μL *X. laevis* egg extract with 2 μg anti-Cdc27 antibody (Santa Cruz, clone AF3.1) bound to 5 μL Protein-A Sepharose (Sigma), and ubiquitination reactions were performed as described^18,75^. Alternatively, Strep-tagged APC and its activator His-Cdh1 were purified from insect cells infected with baculovirus-based expression vectors^76^. In the latter case, 0.015 μM Strep-APC and 0.3 μM His-Cdh1 were first preincubated on ice in 1X TBS (25 mM Tris-HCl [pH 7.5], 50 mM NaCl) for 30 min to activate the APC. The conjugation mixture contained 0.3 μM MBP-E1, 1.5 μM His-UbcH10, 0.13 μM His-Ube2S, 100 μM ubiquitin, 5 μM HA-tagged N-terminus fragment of cyclin B1 (HA-NCB1), and the activated APC/C. A 100 μL reaction was typically performed in 1X reaction buffer (25 mM Tris-HCl [pH 7.5], 50 mM NaCl, 10 mM MgCl_2_) in the presence of ATP regeneration system (3.5 U/mL creatine kinase [Roche], 3.8 mM creatine phosphate disodium [Roche], 0.5 mM ATP disodium salt, 0.5 mM MgCl_2_) for 5 h at 25 °C. Glycerol was added to the ubiquitinated NCB1 conjugates (HA-Ub_n_-NCB1) to a final concentration of 10% (v/v) for storage at −80 °C.

### Preparing “ATP-free” ADP, ADP-proteasome and ADP-HA-Ub_n_-NCB1

Proteasomes were purified in the presence of ATP to preserve their integrity, and ubiquitinated conjugates were generated in the presence of ATP regeneration system. In addition, residual ATP contamination is often found in commercial ADP (EMD Millipore). Depletion of the ATP from these reagents is required to uncouple the effect of Ubp6 deubiquitination to proteasome degradation. To generate “ATP-free” ADP, powder was first dissolved in reaction buffer (50 mM Tris-HCl [pH 7.5], 5 mM MgCl_2_) and adjusted to pH 7.5 using 10 M KOH. The reconstituted ADP was then treated with 20 mM glucose and 0.2 U/μL hexokinase (Sigma) for 1 h at 30 °C. The final ADP concentration in solution was ~0.25 M. To generate ADP-protreasome, proteasomes (final ~0.5 μM) were hexokinase-treated in the same way. For ADP-HA-Ub_n_-NCB1, ubiquitinated conjugates (final ~2.5 μM) were treated similarly but using 40 mM glucose and 0.4 U/μL hexokinase. 10% glycerol (v/v) was added for storage at −80 °C.

### In vitro deubiquitination assays

Purified recombinant Ubp6 variants (120 nM) and ADP-proteasomes (Figs. 2e, 3g: *ubp6*Δ *hul5*Δ i.e., nominally wild-type; Fig. 3g: *rpt1-RK ubp6*Δ *hul5*Δ, 5 nM) were assayed in reaction buffer (50 mM Tris-HCl [pH 7.5], 5 mM MgCl_2,_ 0.5 mM DTT) supplemented with 5 mM “ATP-free” ADP. Proteasome and metalloprotease inhibitors were also supplemented in the reaction, which includes 0.5 mM ATPγS (Santa Cruz), 1.5 μM PS-341 (ApexBio), 7.5 μM MG-262 (Apexbio), 100 μM Epoxomicin (ApexBio), and 10 mM 1,10-phenanthroline (o-PA, Sigma). Ubp6 and ADP-proteasomes were preincubated for 15 min at 30 °C. 200 nM ADP-HA-Ub_n_-NCB1 was then added to the Ubp6-proteasome mixture to initiate the reaction. Samples were subsequently analyzed by SDS-PAGE and immunoblotted using the following antibodies: 1:1,000 HA-HRP (Sigma), 1:50,000 Ubp6^36^, 1:10,000 Rpn13 (this study). All in vitro deubiquitination assays have been independently repeated. Yeast strains used are listed in Supplementary Table 3, and antibody information can be found in Supplementary Table 6.

### In vitro degradation assays

Purified recombinant Ubp6 variants (Fig. 4a, b: 120 nM; Fig. 4c, d: 240 nM) were first incubated with nominally wild-type (Fig. 4a–d: *ubp6*Δ *hul5*Δ ecm29Δ, 30 nM) or mutant (Fig. 4c, d: *rpt1-RK ubp6*Δ *hul5*Δ ecm29Δ, 30 nM) yeast proteasomes for 15 min. Where indicated, Ubp6 was preincubated with 5–10-fold excess of UbVME to induce adduct formation prior to incubation with the proteasome. 200 nM HA-Ub_n_-NCB1 was then added to the Ubp6-proteasome mixture to initiate the reaction. At the indicated time points, 10 μL of the reaction mixture was withdrawn, and the reaction was terminated by adding 2X Laemmli loading buffer and heating at 70 °C for 10 min. Samples were collected and analyzed as above using the following antibodies: 1:1,000 HA-HRP (Sigma), 1:50,000 Ubp6^36^, 1:15,000 Rpn8^24^. All in vitro degradation assays have been independently repeated.

### Yeast strain construction

Standard techniques were used for strain constructions and transformations. Yeast strains built for this study were either prepared by crossing of haploids bearing existing alleles, or by transformation of diploids with gene disruption constructs, followed by sporulation and dissection to retrieve the target haploid. For yeast transformation, a single yeast colony was inoculated into 5 ml of YPD and incubated overnight at 30 °C with shaking. The overnight starter culture was then diluted into 50 mL of YPD (1% bacto-yeast extract, 2% bacto-peptone, 2% dextrose, 50 mg/L adenine, 400 mg/L tryptophan, 500 mg/L uridine) at a cell density of 5 × 10^6^ cells/mL, and the sample was then cultured at 30 °C with shaking at 200 rpm until density reached 2 × 10^7^ cells/mL. Cells were subsequently harvested by spinning at 3000 × *g* for 5 min. The cell pellet was resuspended with 25 mL sterile water and centrifuged again. Cell pellet was then washed in 1 mL of 100 mM lithium acetate (LiAc, Sigma), and resuspended with 100 mM LiAc at 2 × 10^9^ cells/ml. For each transformation, 50 μL of 2 × 10^9^ cells/ml cells were transferred to a new microcentrifuge tube and spun down. The following components were added to the cell pellet in the listed order: 240 μL of PEG MW 3350 (Sigma), 36 μL of 1 M LiAc, 10 μL of 10 mg/mL boiled and pre-chilled single-stranded carrier DNA (Agilent), and 5 μg of desired plasmid DNA of interest. Each sample was vortexed vigorously until the cell pellet was completely resuspended. The transformation mixture was incubated at 30 °C for 30 min, then heat-shocked in a 42 °C water bath for 20 min. The mixture was then spun at 4000 × *g* for 15 s. Lastly, the cell pellet was resuspended in YPD or selective medium, depending on genetic selection to be imposed, and the sample was plated onto corresponding agar plates for 30 °C incubation. A list of plasmids used for yeast transformation can be found in Supplementary Table 7.

### Yeast assays

For plate assays, strains were inoculated into either YPD (1% bacto-yeast extract, 2% bacto-peptone, 2% dextrose, 50 mg/L adenine, 400 mg/L tryptophan, 500 mg/L uridine) or selective media and grown overnight at 30 °C. Cell density was measured, and the cultures were diluted with fresh media to OD_600_ = 0.1. Cultures were allowed to grow at 30 °C for another 3–5 h, until the OD_600_ reached log phase. Cultures were then serially diluted three-fold in media, spotted onto plates with a pin array, and incubated at 30 °C for 3–7 days.

All plates were prepared using media supplemented with 2% agar. Synthetic plating medium consisted of 2% dextrose, 0.67% yeast nitrogen base without amino acids, 12.5 mg/L adenine hemisulfate, 125 mg/L uridine, 50 mg/L phenylalanine, 50 mg/L isoleucine, 75 mg/L valine, 10 mg/L tyrosine, 150 mg/L proline, 25 mL/L glycine, 75 mg/L alanine, 75 mg/L serine, 50 mg/L threonine, 100 mg/L glutamate, 50 mg/L aspartate, 200 mg/L glutamine, 50 mg/L asparagine, 37.5 mg/L histidine, 112.5 mg/L lysine, 37.5 mg/L methionine, 100 mg/L arginine, 100 mg/L leucine, 100 mg/L tryptophan. Certain amino acids were excluded from the mixture according to the test plate formulation.

For the Ub-K-Trp experiment, medium without uridine (2% dextrose, 0.67% yeast nitrogen base without amino acids, 0.5% casamino acids, 50 mg/L adenine hemisulfate, 500 mg/L tryptophan) was used in Supplementary Fig. 7d to select for the transforming plasmid carrying Ubp6 variants with *URA3* marker. Medium in Fig. 3i was prepared similarly, except with the addition of 500 g/L uridine, as these are uracil auxotrophs. Yeast strains used for Ub-K-Trp assay can be found in Supplementary Table 8.

For canavanine experiments (Fig. 3h and Supplementary Fig. 7e), arginine was omitted because it competes with canavanine. For Supplementary Fig. 7e, uridine and tryptophan were additionally omitted (Ubp6 variants with *URA3* maker and ubiquitin overexpression or corresponding control plasmid with *TRP1* marker) where needed to maintain selection. Canavanine was added to a final concentration of 5 μg/mL. For plasmids in which ubiquitin expression is driven by the *CUP1* promoter, media were supplemented with 100 μM copper sulfate. Yeast strains used for canavanine assays can be found in Supplementary Table 9.

At least five transformants were picked 3–4 days post-plating. Each set of plate assays was done with at least three transformants and independently repeated, all yielding equivalent results. Representative images were shown in this study.

For evaluating the Ubp6 noncatalytic effect in vivo, a colony forming assay was used (Fig. 4e, f). Plasmids expressing variants of Ubp6 were transformed into either *ubp6*Δ*rpn4*Δ (sJH185) or *ubp6*Δ (sJH183) yeast strains. Colony formation was scored after incubation at 30 °C for 3–4 days. *RPN4* deletion eliminates the cells’ capability to counteract proteasome stress by upregulating proteasome subunit synthesis. The presence of catalytically inactive *ubp6-C118A* mutation results in cell death due to ubiquitin stress (i.e., ubiquitin depletion resulting from failure of catalytic deubiquitination prior to substrate degradation), together with proteasome stress from degradation inhibition (i.e., the noncatalytic effect). This assay has been independently repeated and yeast strains used for this assay can be found in Supplementary Table 10.

### Reagents

Additional information on commercial reagents used in this study is detailed in Supplementary Table 11.

### Reporting Summary

Further information on research design is available in the Nature Research Reporting Summary linked to this article.

## Supplementary information

Supplementary Information

Description of Additional Supplementary Files

Supplementary Data 1

Reporting Summary

## Data Availability

Plasmids and yeast strains are available upon request. The cryo-EM density maps have been deposited to the Electron Microscopy Data Bank (EMDB) under accession codes: EMD-14082 (si body1), EMD-14083 (si body2), EMD-14084 (si^Rpt1-RK^), EMD-14085 (s2^Rpt1-RK^). The models from this study are available through the Protein Data Bank (PDB) under the following accession codes: 7QO3 (si body1), 7QO4 (si body2), 7QO5 (si^Rpt1-RK^), 7QO6 (s2^Rpt1-RK^). Protein structures from previous publication can be found under PDB accession codes 1VJV (Ubp6 crystal structure)^77^, 6EF3 (26S yeast proteasome bound to ubiquitinated substrate)^78^, 2AYO (USP14 bound to ubiquitin aldehyde)^38^, 6FVX (s5 state)^40^, 6FVV (s3 state)^40^, 6FVU (s2 state)^40^. Hydrogen deuterium exchange mass spectrometry (HDX-MS) kinetic data are provided in Supplementary Data 1. All HDX-MS data have been deposited to the ProteomeXchange Consortium via the PRIDE^74^ partner repository with the dataset identifier PXD029402.  Source data, including raw data and images, are provided with this paper.

## Source data

Source Data Files
