# Supplementary material for: Allosteric control of Ubp6 and the proteasome via a bidirectional switch
Source: Nat Commun. 2022 Feb 11;13:838. doi: 10.1038/s41467-022-28186-y (PMC8837689; doi:10.1038/s41467-022-28186-y)
Supplement: Supplementary file 1 — Supplementary Information [file 41467_2022_28186_MOESM1_ESM.pdf]

Supplementary Information for

A bidirectional switch coordinates allosteric control of Ubp6 and the proteasome

Ka Ying Sharon Hung, Sven Klumpe, Markus R. Eisele, Suzanne Elsasser, Geng Tian, Shuangwu Sun, Jamie A. Moroco, Tat Cheung Cheng, Tapan Joshi, Timo Seibel, Duco Van Dalen, Daniela Schibich, Andre Schwarz, Andreas Ulmer, Ying Lu, Huib Ovaa, John R. Engen, Byung-Hoon Lee*, Till Rudack*, Eri Sakata*, and Daniel Finley*

*Correspondence to: daniel_finley@hms.harvard.edu (D.F.) , eri.sakata@med.uni-goettingen.de (E.S.), till.rudack@rub.de (T.R.), and byung-hoon_lee@dgist.ac.kr. (B.H.L.)

Supplementary file includes:

Supplementary Figs. 1 to 12
Supplementary Tables 1 to 11
Supplementary References

Supplementary figures:

Supplementary Fig. 1 | Image processing of the 26S-Ubp6-UbVS.
Supplementary Fig. 2 | Classification and refinement scheme of the cryo-EM data.
Supplementary Fig. 3 | Cryo-EM reconstructions and fitted structural models of 26S proteasome-Ubp6 complexes.
Supplementary Fig. 4 | Functional elements of Ubp6.
Supplementary Fig. 5 | Hydrogen-deuterium exchange mass spectrometry confirms Ubp6 interaction with the OB domain of Rpt1.
Supplementary Fig. 6 | The blocking loop network of Ubp6.
Supplementary Fig. 7 | Characterization of *ubp6-I329A L330A* and *USP14-V343 L344* mutants.
Supplementary Fig. 8 | An Rpt1 mutant defective in Ubp6 activation.
Supplementary Fig. 9 | Structural comparisons of proteasome conformational states observed in the presence and absence of Ubp6.
Supplementary Fig. 10 | The BL1 and BL2 loops in free Ubp6 clash with ubiquitin density in si proteasomes.
Supplementary Fig. 11 | Detailed view of the conformational change of BL1 and BL2 loops.
Supplementary Fig. 12 | The *Rpt1-S164R T166K* double mutation blocks Ubp6 activation and shifts the ternary complex from the inhibited si-state to the s2-state.

Supplementary tables:

Supplementary Table 1 | Cryo-EM data collection, refinement and validation statistics.
Supplementary Table 2 | Plasmid information for recombinant protein expression.
Supplementary Table 3 | Strains used for biochemical assays of purified yeast proteasomes.
Supplementary Table 4 | Yeast strains used to purify proteasomes for cyro-electron microscopy studies.
Supplementary Table 5 | Hydrogen deuterium exchange mass spectrometry data summary and list of experimental parameters.
Supplementary Table 6 | Antibodies used in this study.
Supplementary Table 7 | Plasmid information for yeast transformation.
Supplementary Table 8 | Yeast strains used for Ub-K-Trp assay.
Supplementary Table 9 | Yeast strains used for canavanine assay.
Supplementary Table 10 | Yeast strains used for *in vivo* Ubp6 noncatalytic assay.
Supplementary Table 11 | Reagents used in this study.

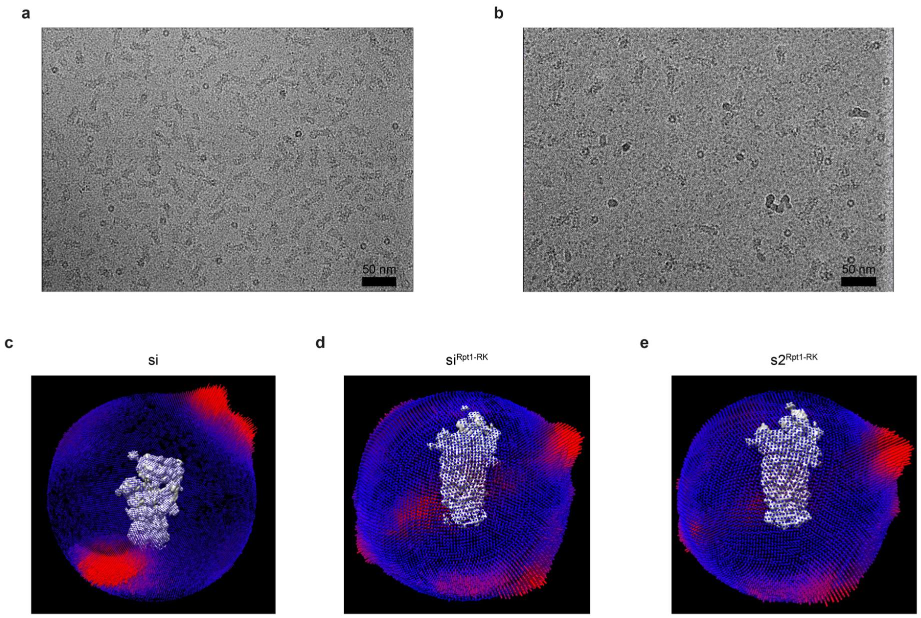

Supplementary Fig. 1 | Image processing of the 26S-Ubp6-UbVS. a-b, Representative cryo-EM micrograph of the (**a**) 26S-Ubp6-UbVS (of a total of 11,233) and (**b**) 26S Rpt1-RK-Ubp6-UbVS (of a total of 9,596) samples. Scale bar represents 50 nm. **C-e,** The angular distribution of the 3D reconstruction of the (**c**) si, (**d**) si$^{Rpt1-RK}$ and (**e**) s2$^{Rpt1-RK}$ proteasome particles.

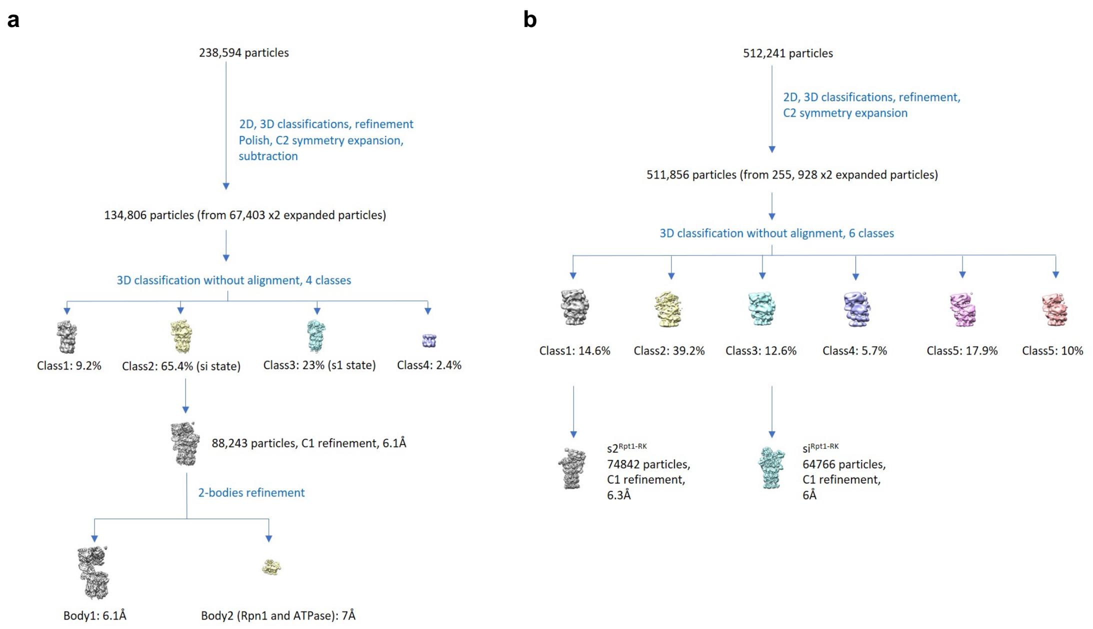

Supplementary Fig. 2 | Classification and refinement scheme of the cryo-EM data. Classification for (**a**) 26S-Ubp6-UbVS and (**b**) 26S Rpt1-RK-Ubp6-UbVS samples.

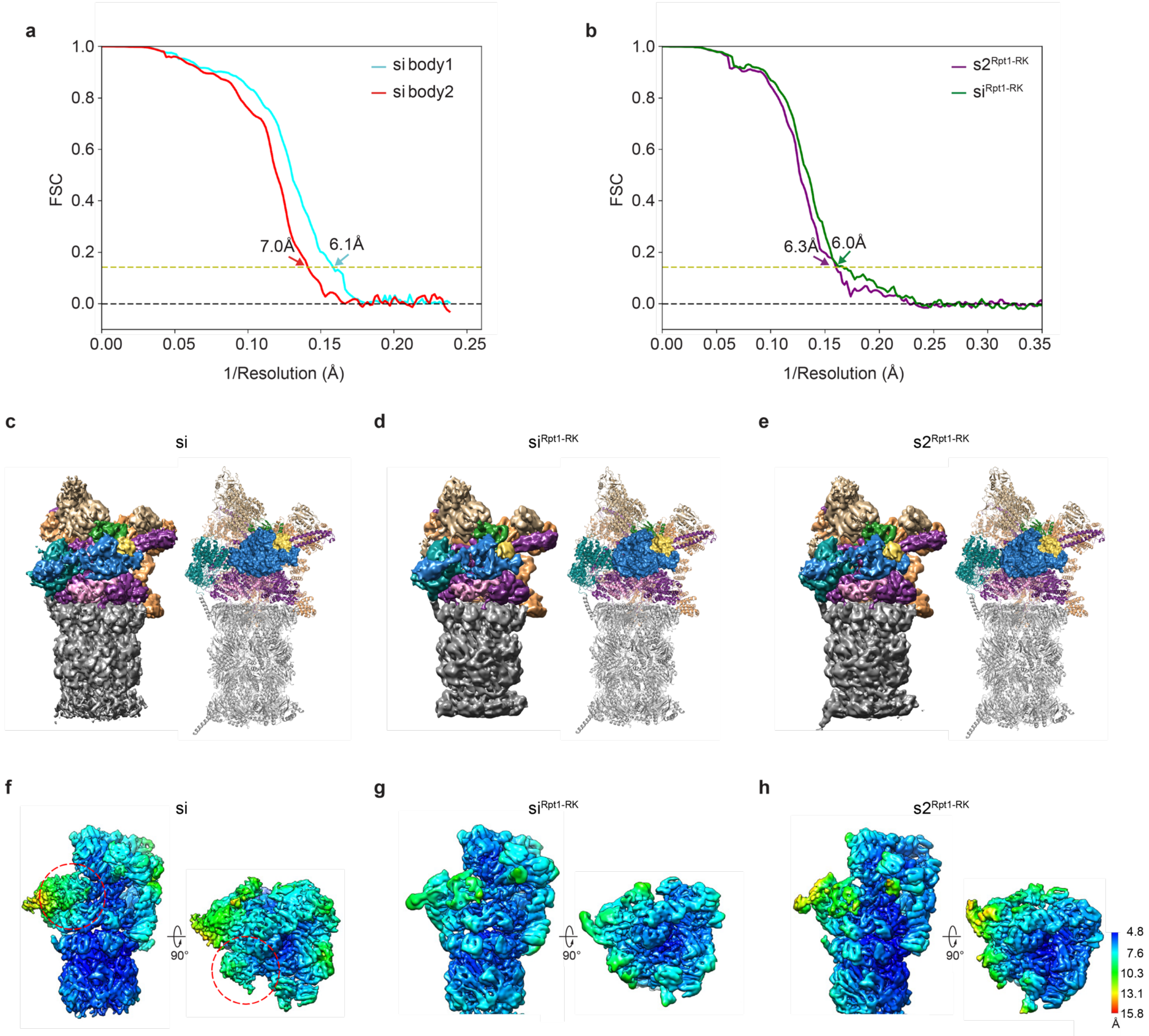

Supplementary Fig. 3 | Cryo-EM reconstructions and fitted structural models of 26S proteasome-Ubp6 complexes. a-b, Fourier shell correlation curves of cryo-EM models in this study. **a**, Resolution of the reconstructions of si proteasomes, body1 (corresponding to the lid and CP) and body2 (corresponding to ATPase, Ubp6 and UbVS). **b**, Resolution of the reconstructions of s2$^{Rpt1-RK}$ and si$^{Rpt1-RK}$ proteasomes. Resolutions are determined on the basis of the gold standard Fourier shell correlation criterion (FSC=0.143, dotted yellow line). See Supplementary Table 1 for additional details. **c-e**, Overview of the cryo-EM reconstructions and the fitted structural model for the si state (**c**, body1, PDB: 7QO3, resolution 6.1 Å; body2, PDB: 7QO4, resolution 7.0 Å), the si$^{Rpt1-RK}$ state (**d**, PDB: 7QO5, resolution 6.0 Å) and s2$^{Rpt1-RK}$ (**e**, PDB: 7QO6, resolution 6.3 Å). The 26S proteasome is colored according to its subunits: Ubp6, blue; ubiquitin, yellow; Rpn11, green; Rpt1, pink; other Rpt subunits, purple; Rpn10 and other base subunits, tan; lid components, light brown; core particle, grey. **f-h**, Local resolution of the (**f**) si, (**g**) si$^{Rpt1-RK}$ and (**h**) s2$^{Rpt1-RK}$ structures is mapped to the reconstruction density isosurfaces as given by the color gradient (angstrom units). Density of the Ubp6 catalytic domain is highlighted by red circle.

a

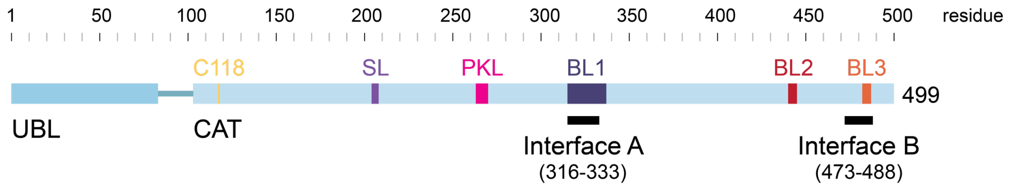

b

	UBP6 residue	UBP6 sequence	USP14 residue	USP14 sequence
Catalytic site	118	C	114	C
SL (Switching Loop)	205-208	YKQQ	195-198	YLQQ
PKL (Pinky Knuckle Loop)	264-270	HISGTTN	278-284	FINQEVK
BL1	316-337	R**FFW**KRSTNKKSK**ILR**KVVFPF	330-351	R**FFY**KEKESVNAK**VLK**DVKFPL
β8a	317-320	FFWK	N/A	N/A
β8b	324-328	NKKSK	N/A	N/A
Ub clash / inhibitory site	317-319	FFW	331-333	FFY
ILR / activation site	329-331	ILR	343-345	VLK
BL2	441-445	ANSES	429-433	RSSSS
BL3	483-487	GGESD	469-473	GGDWH

Supplementary Fig. 4 | Functional elements of Ubp6. a, Linear representation of Ubp6. Ubiquitin-like (UBL) and catalytic (CAT) domains of Ubp6 are represented as blue boxes connected by a linker. Catalytic cysteine C118 and the five key loops are color-coded as in Fig. 1. Amino acid residue numbers of Ubp6 are shown at top. Ubp6-Rpt1 interfaces suggested from cryo-EM data[1] are indicated as black bars at bottom. **b**, Summary of key elements in the catalytic domains of Ubp6 and USP14.

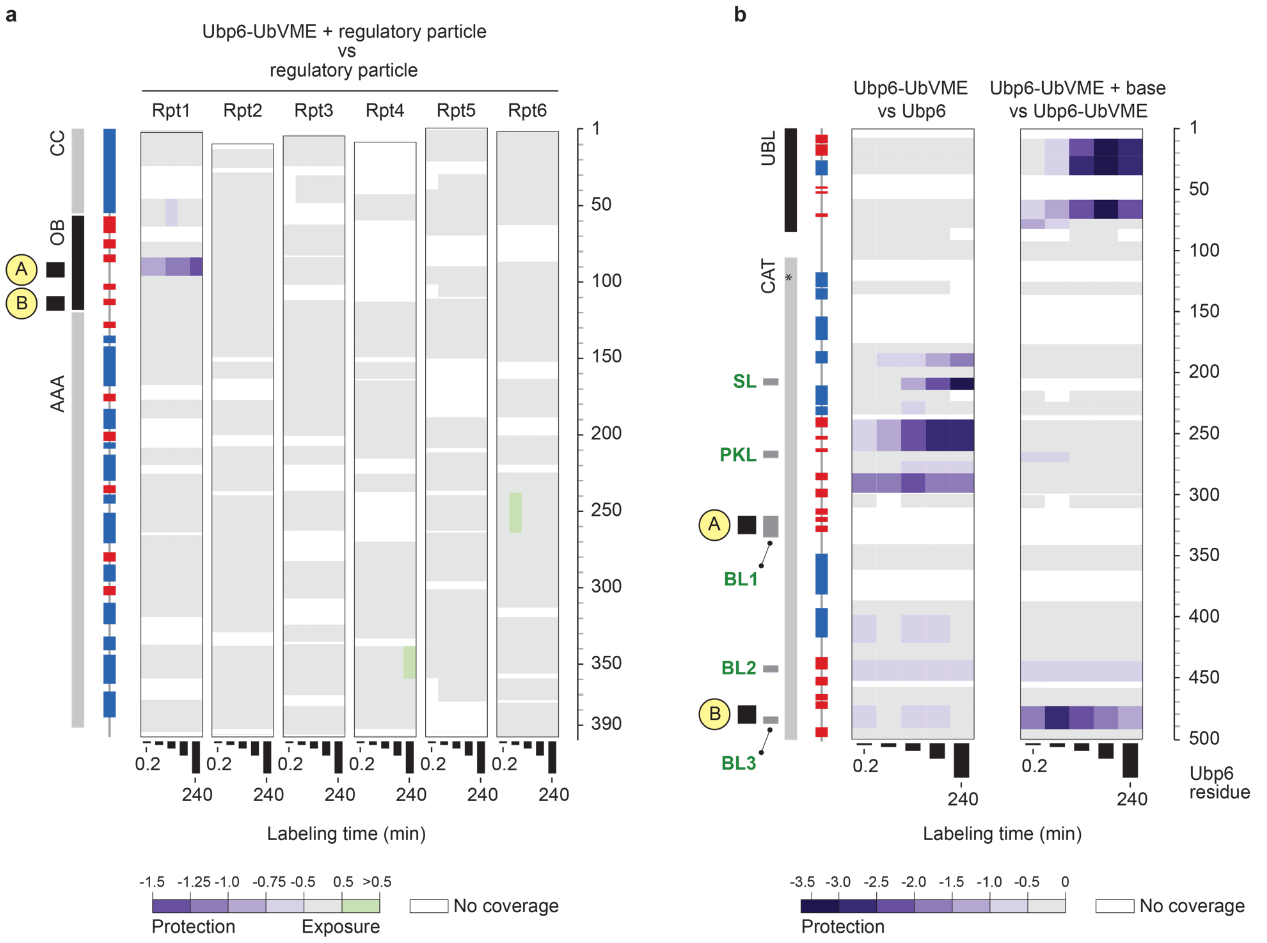

Supplementary Fig. 5 | Hydrogen-deuterium exchange mass spectrometry confirms Ubp6 interaction with the OB domain of Rpt1. a, HDX-MS was performed to localize the Ubp6-Rpt1 interaction. This panel provides the complete data set from which Fig. 1c was abstracted. Deuterium exchange was monitored over four hours at time points of 0.17, 1, 10, 60, 240 min (represented by vertical bars). Deuteration differences of purified *ubp6Δ* regulatory particle (RP) alone versus RP in the presence of 5-fold molar excess of Ubp6-UbVME adduct were measured. Deuterium differences ($D_{RP+Ubp6\text{-}UbVME} – D_{RP\ alone}$) are color-coded according to the scale at bottom, with the strongest shade of purple being most protected (i.e., the least amount of deuterium exchange) in the presence of Ubp6-UbVME. Domain organization at left and residue numbers at right refer to information determined from a master alignment between archaeal Rpt homolog proteasome-activating nucleotidase (PAN) and the six Rpt subunits in *S. cerevisiae*. Α-helices and β-strands are depicted by blue and red boxes respectively. Interfaces A and B are shown as black boxes. Major protection is seen in the OB domain of Rpt1. This protection is unique to Rpt1; no other subunit (Rpt2 -Rpt6) of the ATPase ring shows protection. **b**, Deuterium exchange in Ubp6. Domain organization of Ubp6, recovered from PDB:1WGG for the UBL and from the si structure for CAT, is shown at left, while residue numbers of Ubp6 are shown at right. All key Ubp6 loops are shown in grey boxes. The catalytic residue C118 is indicated by an asterisk. Differences in peptide deuteration over time are color-coded according to the scale at bottom. The left panel shows the differences ($D_{Ubp6\text{-}UbVME} – D_{Ubp6}$) in deuteration of recombinant Ubp6-UbVME adduct versus Ubp6 alone. UbVME-induced protection is localized to a region of the

catalytic domain. The right panel shows the differences ($D_{Ubp6\text{-}UbVME+base}$ – $D_{Ubp6\text{-}UbVME\ alone}$) in deuteration of the recombinant Ubp6-UbVME adduct alone versus in the presence of a 1.6-fold excess of recombinant proteasome base. Differences at each time point are color-coded according to the scale at the bottom. Strong protection within the UBL domain appears to represent its interaction with proteasomal subunit Rpn1[2]. For the CAT domain, only Interface B was shown to be protected; whereas peptides covering Interface A, which contains residues I329 and L330, were not resolved in this analysis. All deuterium uptake values used to generate these difference maps can be found in Supplementary Data 1.

a

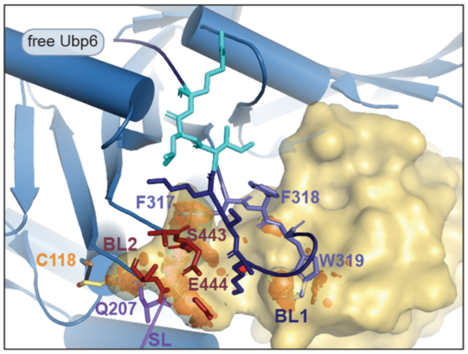

b

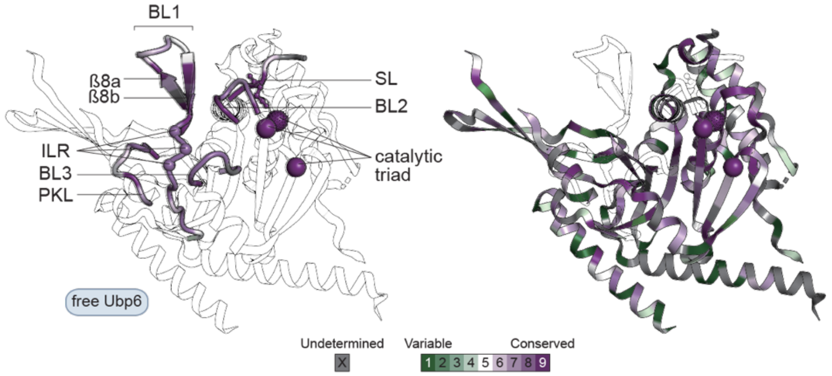

c

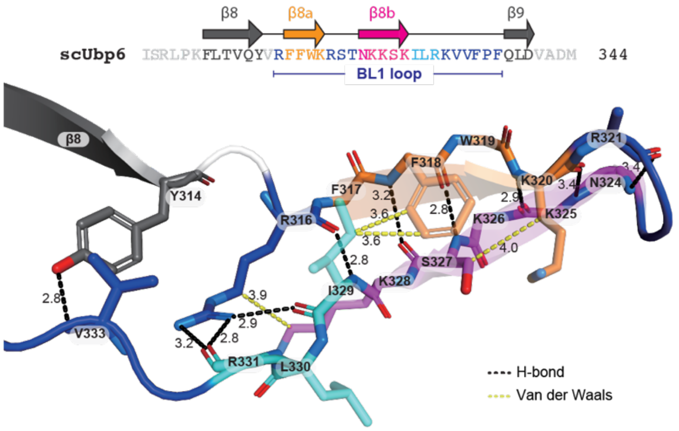

Identified interstrand bonds	β8a side	β8b side	Distance (Å)
β-sheet H-bond	R321 -C=O	N324 -N	3.4
β-sheet H-bond	K320 -C=O	K325 -N	3.4
β-sheet H-bond	K320 -N	K325 -C=O	2.9
β-sheet H-bond	F318 -C=O	S327 -N	2.8
β-sheet H-bond	F318 -N	S327 -C=O	3.2
H-bond	R316 -C=O	I329 -N	2.8
H-bond	R316 -NH1	I329 -C=O	2.9
H-bond	R316 -NH1	R331 -C=O	2.8
H-bond	R316 -NH2	R331 -C=O	3.2
H-bond	Y314 -OH	V333 -C=O	2.8
Van der Waals interaction	K320 -Cβ	S327 -Cβ	4.0
Van der Waals interaction	F318 -Cδ1	I329 -Cγ1	3.6
Van der Waals interaction	F318 -Cε1	I329 -Cγ1	3.6
Van der Waals interaction	R316 -Cδ	K328 -Cε	3.9

d

		B factor of main chain Cα (Å²)	Average of B factors of all atoms within residue (Å²)
Ubp6 CAT domain		20.8 ± 9.0	23.2 ± 10.4
SL	Q207	13.1	15.5 ± 2.3
BL1	F317	14.7	16.5 ± 2.1
	I329	18.4	21.8 ± 5.8
	L330	18.3	18.5 ± 1.9
BL2	S443	17.6	17.9 ± 1.8
	E444	19.1	23.4 ± 5.6

e

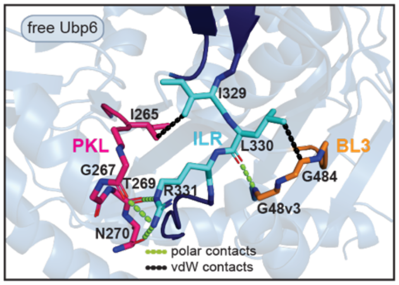

f

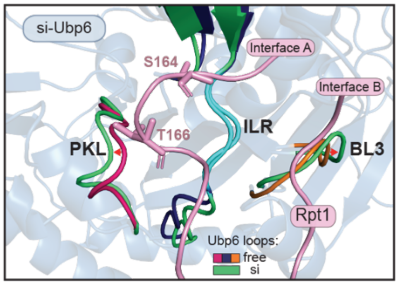

Supplementary Fig. 6 | The blocking loop network of Ubp6. a, Zoomed-in view of ubiquitin-blocking loop clashes. The ubiquitin moiety from USP14-UbAl[3] is superposed onto the free Ubp6 crystal structure. Steric hindrance, shown as embedded orange discs, can be seen between residues F317-W319 of BL1 (slate blue), BL2 (raspberry), Q207 of SL (purple) and ubiquitin (yellow). Specifically, prominent clashes from side chains of F317 and W319 and main chain of F318 in BL1 are evident, as are main chain and side chain clashes from S443 and E444 of BL2. The ILR activation element, which is within BL1, is displayed in cyan. In all panels, the isopeptide bond between the active site cysteine (orange) and C-terminus of ubiquitin (red) is represented in black. **b**, The blocking loop network of Ubp6 is evolutionarily conserved. Multiple sequence alignment of Ubp6 was performed with 50 orthologs from a diverse set of eukaryotes. Conservation analysis was then performed using the ConSurf server with Bayesian algorithm (https://consurf.tau.ac.il), where the output data represents the estimated evolutionary rate as graded conversation scores. Grade 9 (purple) indicates residues that are most conserved, Grade

1 (green) the most variable. For some residues (grey), evolutionary rate cannot be confidently computed (undetermined), for example when an alignment position has fewer than six ungapped amino acids or when a specific alignment interval contains conservation scores that span four or more grades. 91 out of 499 residues fell into the undetermined category. The nine color conservation scores were projected onto the crystal structure of free Ubp6 for visualization (PDB: 1VJV). (left) Evolutionary conservation of the three blocking loops and of BL3 and PKL. Residue Q207, the key ubiquitin-blocking residue of SL, is shown in ball and stick. The catalytic triad (C118, H447, N465) is rendered as spheres with the active site cysteine stippled. The remaining parts of Ubp6 are only outlined. (right) Overall conservation of the catalytic domain other than residues highlighted in panel *a*. Strong conservation is evident near the catalytic triad. **c**, Interactions within and around blocking loop 1 of Ubp6 stabilize the β-hairpin. Zoomed-in view of BL1 in free Ubp6 (PDB: 1VJV). Proposed main chain and side chain contacts between and adjacent to β-strands 8a (F317-K320, orange) and 8b (K324-K328, magenta) are indicated. Nitrogen and oxygen atoms are highlighted in skyblue and in red respectively. Two residues from the ILR element, I329 and L330, are labelled in cyan. Note that the stabilizing interstrand network extends from the ß-hairpin into and beyond the ILR element. A summary of interstrand interactions among residues Y314-V333 in free Ubp6 is given at right. Note that these bond lengths are derived from the 1.7 Å crystal structure of free Ubp6. **d**, Crystallographic B factors for free Ubp6 are consistent with minimal conformational dynamics in the SL loop, BL2, the ILR, and much of BL1 (excepting the distal tip of the hairpin). Apart from the ILR, residues shown in the table are those assigned as key blocking residues. For each residue specified, the B factors of main chain alpha carbon and the average of B factors ± SD of all atoms within the residue are given (PDB: 1VJV). B factor values less than 30 signify a low level of atomic fluctuation within the crystal. The top row gives averaged B factor values for the entire catalytic domain of Ubp6. **e**, Detail of the activation region at the BL1 base. In free Ubp6, the ILR is braced and held in place through multiple contacts with the adjacent PKL and BL3 loops. **f**, Comparison of the PKL-ILR-BL3 support in free Ubp6 (PDB: 1VJV) and complex si. Structures were aligned on the ILR (cyan). Loops from free Ubp6 were superimposed on si-Ubp6.

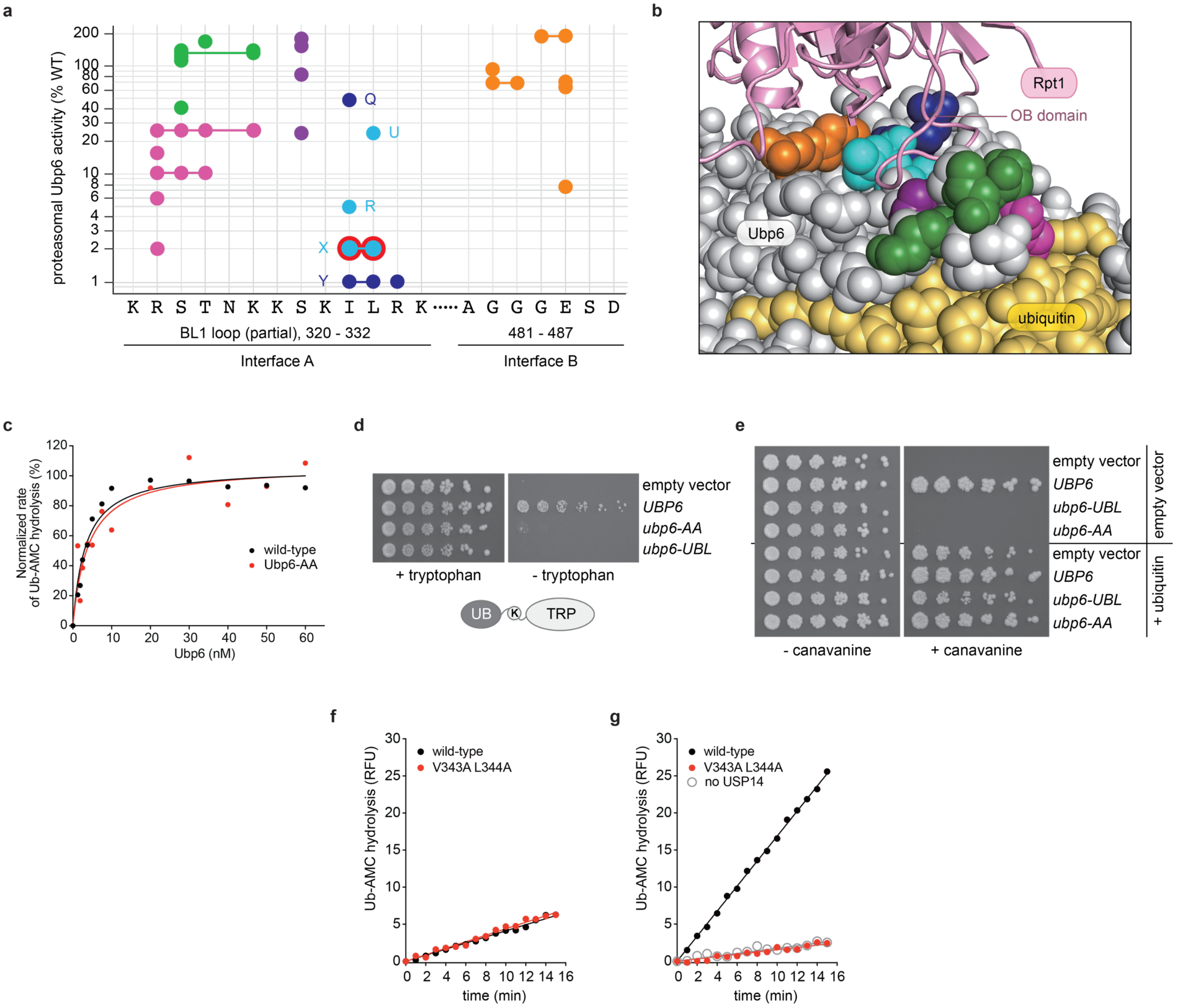

Supplementary Fig. 7 | Characterization of *ubp6-I329A L330A* and *USP14-V343 L344* mutants. a, Proteasome-dependent activity of Ubp6 mutants mapped to the sequences of Interfaces A and B. Mutants color-coded as in Fig. 2a. **b**, Modeled Ubp6-Rpt1 interface with substituted residues color-coded as in Fig. 2a (PDB: this study). **c**, Plot of the same data as Fig. 2f, but with the calculated activity of the Ubp6-AA mutant normalized to that of wild-type. The calculated activity level at 60 nM was taken as the benchmark for normalization. The plot illustrates that the affinity of Ubp6 for the proteasome is only minimally altered by the mutation (apparent K_d of 3.3 nM for wild-type Ubp6 and 4.0 nM for Ubp6-AA), despite the strong defect in catalytic activity. The data were fit by nonlinear regression. The Ubp6-AA data are noisier than those of wild-type due to the low activity of the mutant enzyme. **d**, UB-K-TRP reporter stabilization by the *ubp6-AA* mutant. The UB-K-TRP fusion protein is co-translationally deubiquitinated, resulting in the expression of an unstable C-terminal fragment containing the Trp1 protein with lysine at its endoproteolytically processed N-terminus (K-TRP). K-TRP is efficiently degraded by the proteasome and thus has a short half-life, resulting in growth failure of the *ubp6Δ* strain in the absence of tryptophan. WT Ubp6 can restore growth on media lacking tryptophan by rescuing the reporter from degradation. In this assay, *ubp6Δ* mutant carrying an integrated UB-K-TRP reporter

was transformed with plasmids expressing Ubp6 variants as indicated**.** Yeast cells were serially diluted, plated on media containing or lacking tryptophan, and incubated at 30°C for 3-7 days. **E**, Canavanine sensitivity test. Canavanine is an arginine analog that can be incorporated into newly synthesized proteins, thus impairing protein folding. *Ubp6Δ* mutants are sensitive to canavanine, a phenotype that can result from the inability of cells to handle the increased burden of misfolded protein on proteasomes. In this assay, *ubp6Δ* mutants were transformed with plasmids expressing the indicated variants of *UBP6*. Transformants were serially diluted and transferred to agar plates containing 100 μM $CuSO_4$ in the presence or absence of canavanine at 1.5 μg/mL. Plates were incubated at 30°C for 3-7 days. The canavanine-sensitivity of *ubp6* mutants can be suppressed by ubiquitin overexpression: ubiquitin expression is driven by the *CUP1* promoter and induced by supplemented $CuSO_4$. *UBP6*, wild-type UBP6; *UBL,* UBL domain of Ubp6. **f-g**, The data shown here were used to generate Fig. 2e. **f**, Basal USP14 activity. Ub-AMC hydrolysis assay of wild-type USP14 (1 μM) in its free form, together with the V343A L344A double mutant. **g**, Proteasomal activation of deubiquitination by USP14. Ub-AMC hydrolysis assay of WT and mutant USP14 (8 nM) in the presence of human proteasome (1 nM) pretreated with ubiquitin vinyl sulfone (UbVS) to eliminate UCH-L5 activity. “No USP14,” UbVS-treated human proteasome alone. Source data are provided as a Source Data file.

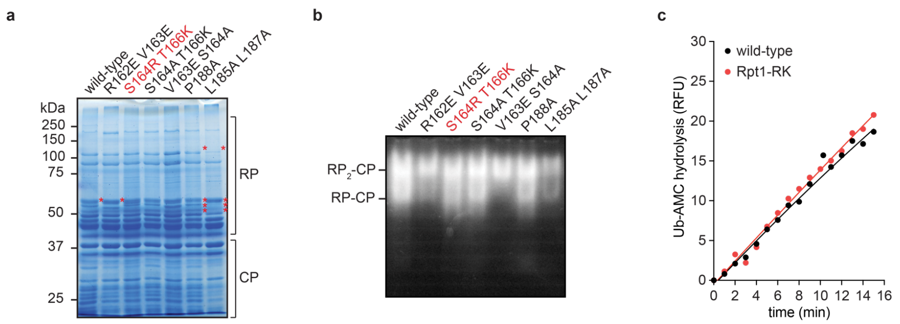

Supplementary Fig. 8 | An Rpt1 mutant defective in Ubp6 activation. **a**, Rpt1-RK mutant proteasomes (S164R T166K) do not exhibit assembly defects**.** Coomassie-blue-stained SDS-PAGE gel showing the subunit composition of purified proteasomes from yeast strains bearing the indicated *rpt1* mutations. The mutant proteasome of interest, Rpt1-RK, shows an electrophoretic profile that is indistinguishable from wild-type. Protein bands reflecting altered subunit composition in other mutants are marked by asterisks. **b**, Purified proteasome complexes were resolved by 3.5% native PAGE, and active species were visualized by an in-gel Suc-LLVY-AMC assay. RP_2-CP and RP-CP are doubly and singly-capped proteasomes, respectively. Proteasome from *rpt1-RK* mutant shows a profile comparable to that from wild-type. Proteasome assembly is perturbed in samples from *rpt1-R162E-V163E*, *rpt-V163E-S164A*, and *rpt1-L185A-L187A* mutant strains. **c**, Time course showing the basal Ub-AMC hydrolytic activity of proteasomes purified from *ubp6Δ hul5Δ* (nominally wild-type) or *rpt1-RK ubp6Δ hul5Δ* strains. 1 μM Ub-AMC was used in this assay. Ubp6-independent “background” hydrolytic activity shown is comparable between the two proteasome samples. Source data are provided as a Source Data file.

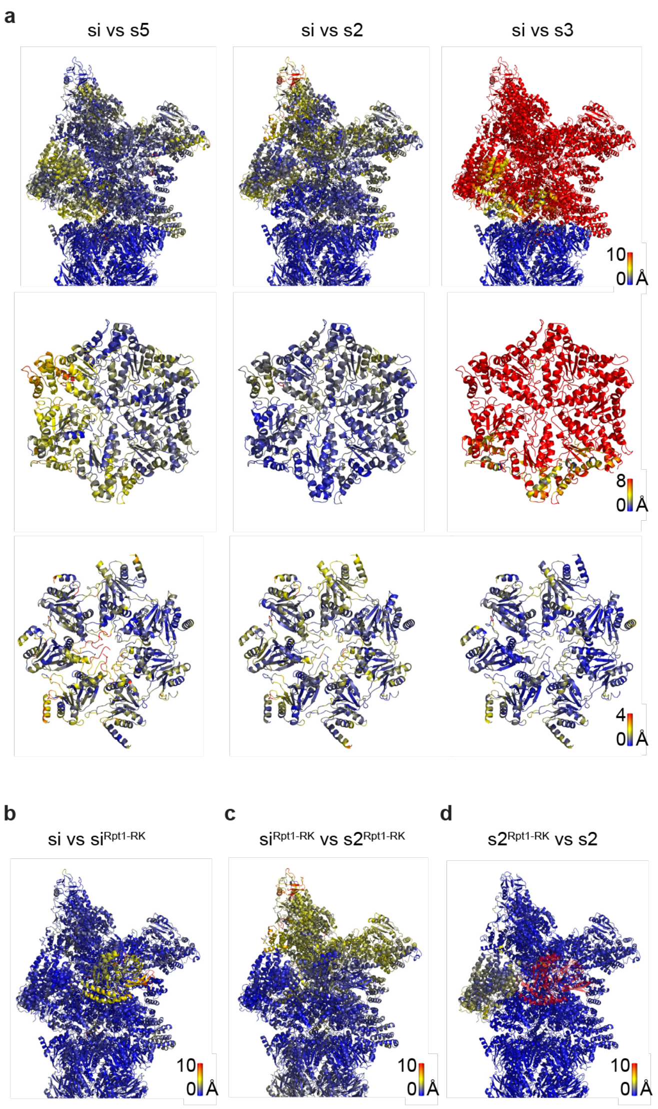

Supplementary Fig. 9 | Structural comparisons of proteasome conformational states observed in the presence and absence of Ubp6. All comparisons shown are visualized by a coloring scheme based on the residuewise root mean square deviation (RMSD). States that are compared are aligned with respect to the CP. The RMSD of each atom is calculated and the RMSD values of all atoms in a given residue are averaged. Then the residues are colored based on the spectral distribution of averaged residuewise RMSD. Residues with small RMSD suggest high similarity between compared structures and are shown in blue, while those with large RMSD, suggesting low similarity between compared structures, are shown in red. **a**, Structural differences between si and s5, s2, or s3 proteasomes. Shown here are the si proteasomes (PDB: 7QO3, 7QO4), which is used as the reference for comparison. Si-state proteasomes combine structural elements of previously reported proteasomal states without bound Ubp6. The RP of si is most similar to s5 (PDB: 6FVX), the ATPase to s2 (PDB: 6FVU), and the gate to s3 (PDB: 6FVV)[4]. **b**, Structural differences between si (PDB: 7QO3, 7QO4) and si$^{Rpt1\text{-}RK}$ (PDB: 7QO5). Shown is si. The Rpt1-RK mutations evoke substantial differences only in Ubp6 positioning; for si state proteasomes, the overall proteasomal structure remains unaffected. **c**, Structural differences between si$^{Rpt1\text{-}RK}$ (PDB: 7QO5) and s2$^{Rpt1\text{-}RK}$ (PDB: 7QO6). Shown is si$^{Rpt1\text{-}RK}$. The two identified states of the Rpt1-RK mutant are almost identical to each other with respect to Ubp6 positioning.

d, Structural differences between the Ubp6-containing $s2^{Rpt1\text{-}RK}$ and the Ubp6-free s2 (PDB: 6FVU)[4]. Shown is $s2^{Rpt1\text{-}RK}$. Ubp6 binding per se does not evoke substantial structural differences for this mutant. The slight differences indicated by the yellow coloring of Rpn1 are due to Rpn1's lower local resolution.

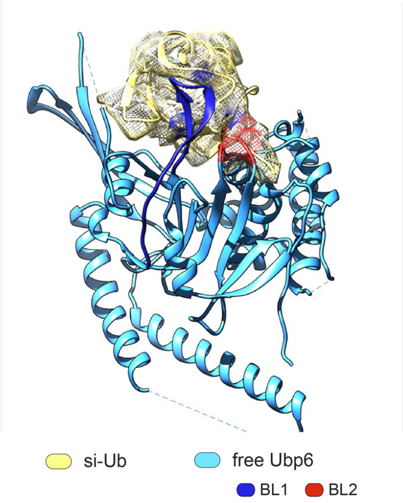

Supplementary Fig. 10 | The BL1 and BL2 loops in free Ubp6 clash with ubiquitin density in si proteasomes. Ubiquitin of the si structure is modelled onto free Ubp6 (PDB: 1VJV). EM density within 2 Å from the model of the BL1 and BL2 loops is colored in blue and red, respectively.

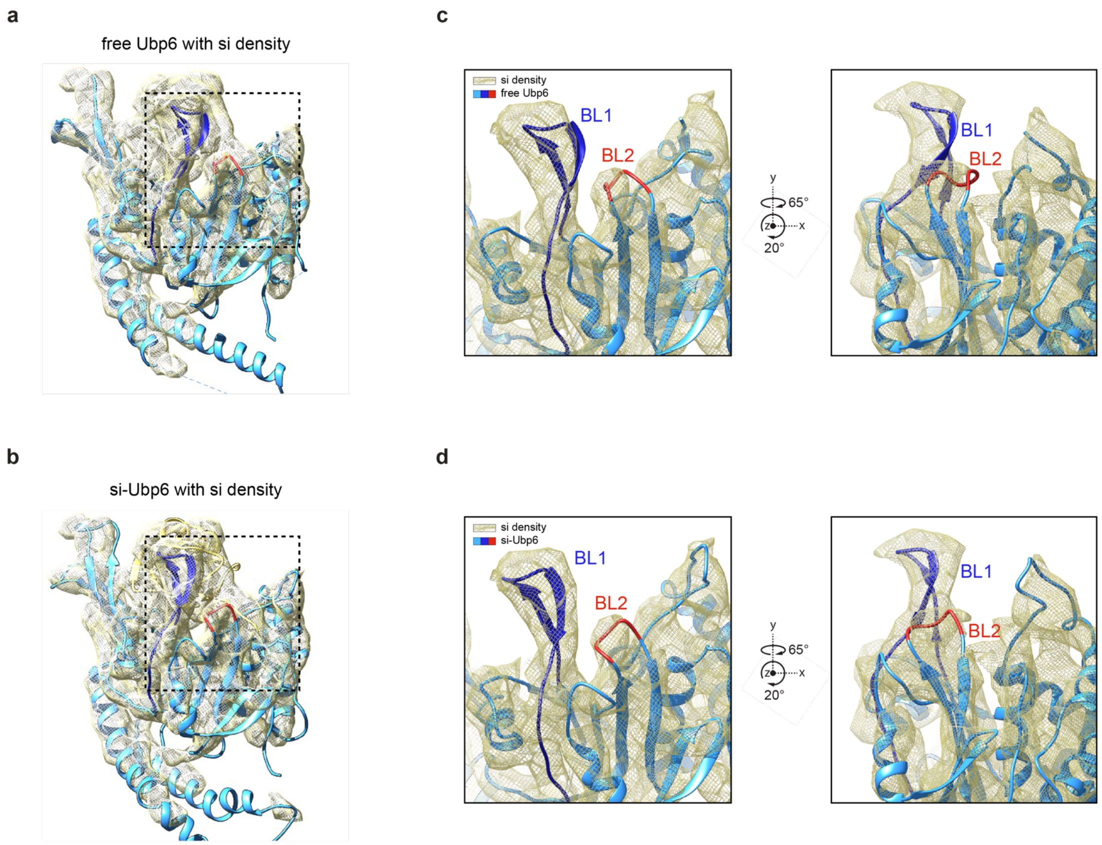

Supplementary Fig. 11 | Detailed view of the conformational change of BL1 and BL2 loops. **a**, Segmented cryo-EM density map of the Ubp6-Ub in the si state proteasome (PDB: 7QO4) is shown with free Ubp6 from the X-ray crystal structure (PDB: 1VJV). **b**, Corresponding model of Ubp6 in si proteasomes. **c**, Detailed zoomed-in view of the conformational change of the boxed region. Segmented Ubp6 cryo-EM density of the si state is shown with free Ubp6 (PDB:1VJV) and (**d**) the corresponding model of Ubp6 in si proteasomes. Ubp6 structures excluding BL1(316-337), BL2(441-445) and peripheral two helices (345-416) were fitted into the si density. BL1 and BL2 are highlighted in blue and red respectively. The BL1 and BL2 loops of free Ubp6 do not fit well with the Ubp6 cryo-EM density from the si proteasome.

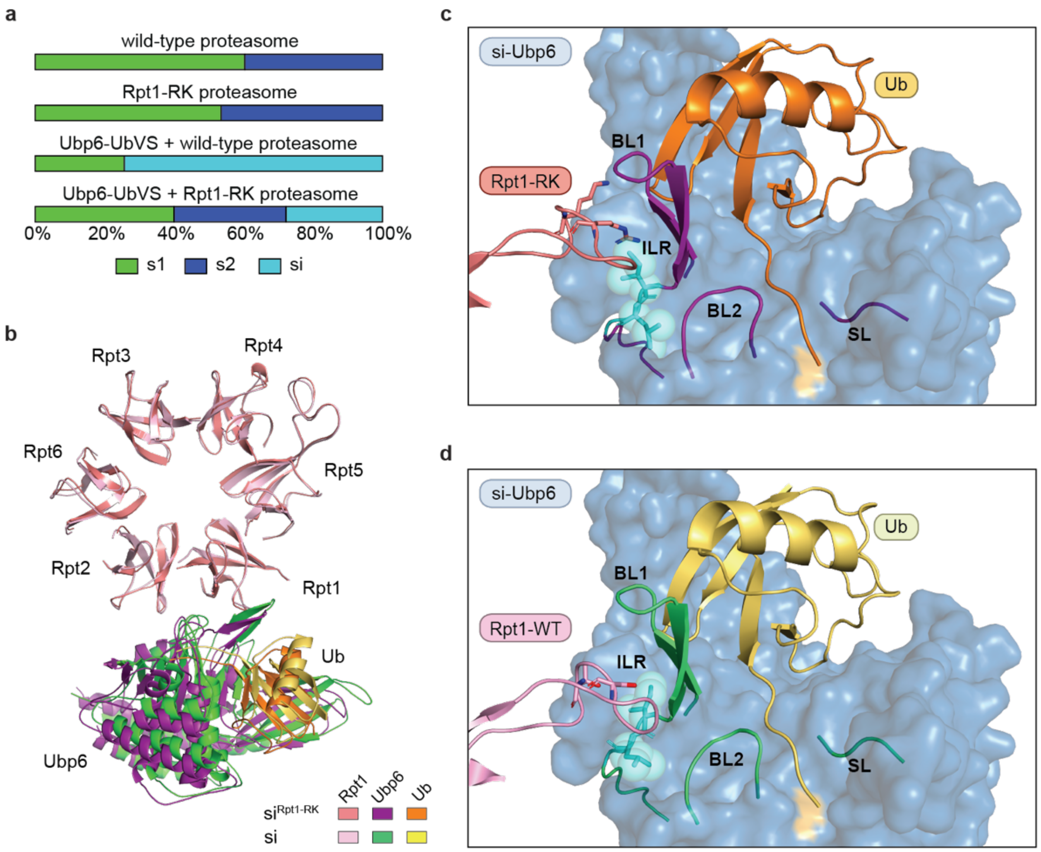

Supplementary Fig. 12 | The *Rpt1-S164R T166K* double mutation blocks Ubp6 activation and shifts the ternary complex from the inhibited si-state to the s2-state. a, Effect of the *rpt1-RK* mutation on the proteasome state distribution. **b**, The *Rpt1-RK* mutation evokes a repositioning of Ubp6-UbVS relative to the proteasome. The si (PDB: 7QO4) and $si^{Rpt1\text{-}RK}$ (PDB: 7QO5) complexes were aligned by their OB rings. **c**, Zoomed-in view of the contact interface between the activation loop of Rpt1-RK (salmon) and BL1 (purple) of Ubp6 in the $si^{Rpt1\text{-}RK}$ proteasome. The positively charged bulky side chains of residues R164 and K166 on the Rpt1 mutant result in less extensive contacts between the activation loop and the ILR. **d**, Zoomed-in view of the contact interface between the activation loop of wild-type Rpt1 (pink) and the ILR element (cyan) within BL1 of Ubp6.

Supplementary Table 1 | Cryo-EM data collection, refinement and validation statistics

	**si (body1)** (EMD-14082) (PDB: 7QO3)	**si (body2)** (EMD-14083) (PDB: 7QO4)	**$si^{Rpt1-RK}$** (EMD-14084) (PDB: 7QO5)	**$s2^{Rpt1-RK}$** (EMD-14085) (PDB: 7QO6)
**Data collection and processing**				
Sample	26S-Ubp6-UbVS		26S Rpt1-RK-Ubp6-UbVS	
Microscope	Titan Krios		Titan Krios	
Detector	K3		K2	
Magnification	22500		18000	
Voltage (kV)	300		300	
Electron exposure (e–/Å^2)	60		35	
Defocus range (µm)	-1.0 to -2.5		-1.8 to -3.0	
Pixel size (Å)	2.10		1.38	
Symmetry imposed	C1		C1	
Micrographs collected (no.)	11,233		9,596	
Initial particle images (no.)	238,594		512,241	
Final particle images (no.)	88,243		64,766	74,842
Map resolution (Å)	6.1	7.0	6.0	6.3
FSC threshold	0.143	0.143	0.143	0.143
Map resolution range (Å)	4.3–12	6–20.8	4.8–17.8	4.6–15
**Refinement**				
Initial model used (PDB code)	6FVX (Lid)<br>6FVV (CP)	6FVU (ATPase)<br>1VJV (Ubp6)<br>2AYO (UbVS)	si	6FVU<br>1VJV (Ubp6)<br>2AYO (UbVS)
Model resolution (Å)	7.1	8.1	7.5	8.6
FSC threshold	0.5	0.5	0.5	0.5
Map sharpening *B* factor (Å^2)	-306	-520	-263	-304
Model composition				
Non-hydrogen atoms	90,304	22,546	112,857	112,954
Protein residues	11,480	2,831	14,311	14,323
Ligands	0	12	12	12
R.M.S. deviations				
Bond lengths (Å)	0.014	0.011	0.035	0.008
Bond angles (°)	1.996	1.887	2.849	1.527
Validation				
MolProbity score	2.33	2.15	2.52	2.57
Clashscore	11.62	7.09	8.78	11.02
Poor rotamers (%)	2.79	1.94	6.29	6.90
Ramachandran plot				
Favored (%)	93.80	90.46	93.19	94.52
Allowed (%)	5.42	8.19	5.78	4.64
Disallowed (%)	0.78	1.35	1.03	0.84

Supplementary Table 2 | Plasmid information for recombinant protein expression.

Dashes indicate information is not applicable.

Note that all plasmids in this table encode Ubp6 in N-terminally tagged form.

Plasmid Information		Antibiotic Resistance	Tag	Reference	Figure(s)
pT553	pET15b-Ubp6-WT	Ampicillin	His	This study	2a: wild-type
pSH101	pET15b-Ubp6-R321W	Ampicillin	His	This study	2a: Mutant E
pSH102	pET15b-Ubp6-S322Q	Ampicillin	His	This study	2a: Mutant J
pSH103	pET15b-Ubp6-T323W	Ampicillin	His	This study	2a: Mutant K
pSH104	pET15b-Ubp6-K325Q	Ampicillin	His	This study	2a: Mutant L
pSH105	pET15b-Ubp6-S327R	Ampicillin	His	This study	2a: Mutant P
pSH106	pET15b-Ubp6-R321A-S322K-T323A	Ampicillin	His	This study	2a: Mutant D
pSH107	pET15b-Ubp6-S322Q-K325Q	Ampicillin	His	This study	2a: Mutant I
pSH108	pET15b-Ubp6-R321A-S322Q-T323A-K325Q	Ampicillin	His	This study	2a: Mutant C
pSH109	pET15b-Ubp6-I329A-L330A	Ampicillin	His	This study	2a: Mutant X
pSH110	pET15b-Ubp6-I329G-L330G	Ampicillin	His	This study	2a: Mutant Y
pSH111	pET15b-Ubp6-G482 del	Ampicillin	His	This study	2a: Mutant B’
pSH112	pET15b-Ubp6-G482 G483 del	Ampicillin	His	This study	2a: Mutant A’
pSH113	pET15b-Ubp6-G484 E485 del	Ampicillin	His	This study	2a: Mutant C’
pSH114	pET15b-Ubp6-E485G	Ampicillin	His	This study	2a: Mutant E’
pSH115	pET15b-Ubp6-E485W	Ampicillin	His	This study	2a: Mutant F’
pSH116	pET15b-Ubp6-E485K	Ampicillin	His	This study	2a: Mutant D’
pSH117	pET15b-Ubp6-R321A	Ampicillin	His	This study	2a: Mutant B
pSH118	pET15b-Ubp6-S322E	Ampicillin	His	This study	2a: Mutant H
pSH119	pET15b-Ubp6-S322W	Ampicillin	His	This study	2a: Mutant G
pSH120	pET15b-Ubp6-S327A	Ampicillin	His	This study	2a: Mutant O
pSH121	pET15b-Ubp6-S327L	Ampicillin	His	This study	2a: Mutant N
pSH122	pET15b-Ubp6-S327Q	Ampicillin	His	This study	2a: Mutant M
pSH123	pET15b-Ubp6-I329A	Ampicillin	His	This study	2a: Mutant R
pSH124	pET15b-Ubp6-L330A	Ampicillin	His	This study	2a: Mutant U
pSH125	pET15b-Ubp6-S327R-E485W	Ampicillin	His	This study	2a: Mutant G’
pSH126	pET15b-Ubp6-R321E	Ampicillin	His	This study	2a: Mutant A
pSH127	pET15b-Ubp6-S322K	Ampicillin	His	This study	2a: Mutant F
pSH128	pET15b-Ubp6-I329V	Ampicillin	His	This study	2a: Mutant Q
pSH129	pET15b-Ubp6-L330D	Ampicillin	His	This study	2a: Mutant T
pSH130	pET15b-Ubp6-L330E	Ampicillin	His	This study	2a: Mutant S
pSH131	pET15b-Ubp6-R331E	Ampicillin	His	This study	2a: Mutant W
pSH133	pET15b-Ubp6-R331A	Ampicillin	His	This study	2a: Mutant V
T562	pGEX-hUSP14-WT	Ampicillin	GST	This study	2f, Supplementary Fig. 7f, g
pSH134	pGEX-hUSP14-V343A-L344A	Ampicillin	GST	This study	2f, Supplementary Fig. 7f, g
–	pCOLA-1 (FLAG-Rpt1, Rpt2, His6-Rpt3, Rpt4, Rpt5, Rpt6)	Kanamycin	FLAG-Rpt1	[5]	Supplementary Fig. 2b
–	pETDuet-1 (Rpn1, Rpn2, Rpn13)	Ampicillin	–	[5]	Supplementary Fig. 2b
–	pACYCDuet-1 (Nas2, Nas6, Hsm3, Rpn14)	Chloramphenicol	–	[5]	Supplementary Fig. 2b

Supplementary Table 3 | Strains used for biochemical assays of purified yeast proteasomes.

The strains in this table are isogenic to SUB62[6], which has the genotype *lys2-801 leu2-3,2-112 ura3-52 his3-∆200 trp1-1*.

Dashes in the table indicate yeast strains bearing no plasmids.

Yeast Strain	Relevant genotype	Host strain	Figure(s)
SY421	*MATα RPN11-TEV-ProA::HIS3 ubp6::URA3*	–	1c, Supplementary Fig. 2b
SYT1177	*MATa RPN11-TEV-ProA::NAT hul5::KAN ubp6::HIS3*	–	2a, Supplementary Fig. 4a
SY1857	*MATa RPN11-TEV-ProA::HIS3 hul5::KAN ubp6::URA3*	–	2b, 3d, Supplementary Fig. 4c, 5e
SY1851	*MATa RPN11-TEV-ProA::HIS3 hul5::KAN ubp6::URA3 rpt1-S164R-T166K::NAT*	–	3d, Supplementary Fig. 5e
SY1853	*MATa RPN11-TEV-ProA::HIS3 hul5::KAN ubp6::URA3 ecm29::TRP1*	–	2d, 3e, 4a-b
SY1847	*MATa RPN11-TEV-ProA::HIS3 hul5::KAN ubp6::URA3 ecm29::TRP1 rpt1-S164R-T166K::NAT*	–	3e, 4c-d
SSW129	*MATα RPN11-ProA-TEV::HIS3 pCUP1-Ub-K-TRP1::HGR RPT1(WT)::NAT*	–	3a, 3h, 3i
SSW178	*MATα RPN11-ProA-TEV::HIS3 pCUP1-Ub-K-TRP1::HGR rpt1-R162E-V163E::NAT*	–	3a, Supplementary Fig. 5a, b
SSW184	*MATα RPN11-ProA-TEV::HIS3 pCUP1-Ub-K-TRP1::HGR rpt1-V163E-S164A::NAT*	–	3a, Supplementary Fig. 5a, b
SSW182	*MATα RPN11-ProA-TEV::HIS3 pCUP1-Ub-K-TRP1::HGR rpt1-S164A-T166K::NAT*	–	3a, Supplementary Fig. 5a-c
SSW180	*MATα RPN11-ProA-TEV::HIS3 pCUP1-Ub-K-TRP1::HGR rpt1-S164R-T166K::NAT*	–	3a, Supplementary Fig. 5a-c
SSW188	*MATα RPN11-ProA-TEV::HIS3 pCUP1-Ub-K-TRP1::HGR rpt1-L185A-L187A::NAT*	–	3a, Supplementary Fig. 5a-c
SSW186	*MATα RPN11-ProA-TEV::HIS3 pCUP1-Ub-K-TRP1::HGR rpt1-P188A::NAT*	–	3a, Supplementary Fig. 5a-c

Supplementary Table 4 | Yeast strains used to purify proteasomes for cryo-electron microscopy studies.

The strains in this table are isogenic to W303 (*leu2-3,112 trp1-1 can1-100 ura3-1 ade2-1 his3-11,15*) from Yeast Genetic Stock Center (Berkeley, CA).

Where applicable, superscripted numbers reference the origin of the strain.

Yeast Strain	Relevant genotype	Figure(s)
YYS40[7]	*MATa RPN11-3xFLAG-HIS3*	1, 2, 3, 5, Supplementary Fig. 1, 4, 7, 8, 9
SSW234	*MATa RPN11-3xFLAG-HIS3 rpt1-S164R-T166K::NAT*	Supplementary Fig. 1, 7, 8

Supplementary Table 5 | Hydrogen deuterium exchange mass spectrometry data summary and list of experimental parameters.

**Data Set**	Measure uptake in RP<br>(Fig. 1d; Supplementary Fig. 5a)	Measure uptake in Ubp6<br>(Supplementary Fig. 5b)
**States analyzed**	1. RP alone<br>2. RP + Ubp6UbVME (1:5)	3. Ubp6 alone<br>4. Ubp6UbVME alone<br>5. Ubp6UbVME + base (1:2)
**HDX reaction details [a]**	Final D_2O concentration = 92.2%, pH_{read} = 7.15, 21 °C	
**HDX time course**	0.167, 1, 10, 60, 240 minutes	
**HDX controls**	7 undeuterated per replicate experiment	6-8 Ubp6+/-UbVMe<br>undeuterated per replicate experiment
**Back-exchange**	30-35%	
**Filtering parameters**	0.3 products/amino acid; 2 consecutive products; 10 ppm error; 4/7 file threshold	0.3 products/amino acid; 1 consecutive product; 10 ppm error; 4/6 or 5/8 file threshold
**Number of peptides [b]**	Rpt1: 45 followed; 62 identified<br>Rpt2: 42 followed; 54 identified<br>Rpt3: 42 followed; 68 identified<br>Rpt4: 32 followed; 42 identified<br>Rpt5: 48 followed; 62 identified<br>Rpt6: 53 followed; 63 identified	71 followed; 87 identified
**Sequence coverage [b]**	Rpt1: 69.2%; Rpt2: 77.1%<br>Rpt3: 80.1%; Rpt4: 56.8%<br>Rpt5: 74.9%; Rpt6: 83.7%	73.3%
**Average peptide length/ Redundancy [b]**	Rpt1: 15.7; 2.15; Rpt2: 16.4; 2.03<br>Rpt3: 14.4; 1.77; Rpt4: 15.4; 1.99<br>Rpt5: 13.7; 2.02; Rpt6: 16.0; 2.50	13.6; 2.53
**Replicates**	2 technical per state	
**Repeatability [c]**	+/- 0.20 relative Da	
**Meaningful Differences**	> 0.5 Da	

[a] 12-fold dilution with labeling buffer (10 mM HEPES-NaOD [pD 7.5], 50 mM NaCl, 50 mM KCl, 5 mM $MgCl_2$, 0.5 mM EDTA, 0.5 mM ATP, 1 mM DTT, 10% glycerol, 99.9% D_2O). 1:1 dilution with quench buffer (0.8 M guanidinium chloride, 0.8% [v/v] formic acid, H_2O, pH 2.0)

[b] Averages of separately processed replicates

[c] No statistical tests were applied to the HDX MS measurements. Rather, based on measurements of mean methodological error (+/- 0.14 Da)[8], we chose a value (+/- 0.50 Da) well above that as the threshold for calling differences in relative deuterium incorporation measurements meaningful. See also explanations of this methodology in Engen JR and Wales TE. (2015)[9].

Supplementary Table 6 | Antibodies used in this study.

Antibody		Working Dilution	Source	Reference
HA epitope YPYDVPDYA, clone 3F10	HRP-conjugated	1:1,000	Sigma	12013819001
Ubp6	Rabbit polyclonal	1:50,000	Finley lab	[2]
Rpn13	Rabbit polyclonal	1:10,000	Finley lab	This study
Rpn8	Rabbit polyclonal	1:15,000	Finley lab	[10]
Cdc27, clone AF3.1	Mouse monoclonal	N/A	Santa Cruz	sc-9972

Supplementary Table 7 | Plasmid information for yeast transformation.

Plasmid Information		Backbone	Reference	Figure(s)
YCplac33	CEN4/URA3, empty vector	n.a.	[11]	4e, 4f, Supplementary Fig. 7e, 7d
pJH80	UBP6-WT	YPplac33	[12]	4e, 4f, Supplementary Fig. 7e, 7d
pJH81	ubp6-C118A	YPplac33	[12]	4e, 4f
pySH01	ubp6-I329A-L330A	YPplac33	This study	4e, Supplementary Fig. 7e, 7d
pySH04	ubp6-C118A-I329A-L330A	YPplac33	This study	4e
pySH07	ubp6-UBL	YPplac33	This study	Supplementary Fig. 7e, 7d
YEp46Δ	2μ/TRP1, empty vector	n.a.	[13]	Supplementary Fig. 7e
YEp96	pCUP1-Ub	YEp46Δ	[13]	Supplementary Fig. 7e

Supplementary Table 8 | Yeast strains used for Ub-K-Trp assay.

The strains in this table are isogenic to SUB62 (as above, [6]).

Dashes in the table indicate yeast strains bearing no plasmids.

Where applicable, superscripted number references the origin of the yeast strain.

Yeast Strain	Relevant genotype	Host strain	Figure(s)
sJH138[12]	*MATa pCUP1-Ub-K-TRP1::NAT ubp6::KAN*	–	–
ySH29	*MATa pCUP1-Ub-K-TRP1::NAT ubp6::KAN (YCplac33 [CEN4/URA3])*	sJH138	Supplementary Fig. 7d
ySH30	*MATa pCUP1-Ub-K-TRP1::NAT ubp6::KAN (pJH80: UBP6-WT [CEN4/URA3])*	sJH138	Supplementary Fig. 7d
ySH31	*MATa pCUP1-Ub-K-TRP1::NAT ubp6::KAN (pySH01: ubp6-I329A-L330A [CEN4/URA3])*	sJH138	Supplementary Fig. 7d
ySH53	*MATa pCUP1-Ub-K-TRP1::NAT ubp6::KAN (pySH07: ubp6-UBL [CEN4/URA3])*	sJH138	Supplementary Fig. 7d
YTS119	*MATa pCUP1-Ub-K-TRP1::HGR RPT1(WT)::NAT*	–	3i
YTS113	*MATa pCUP1-Ub-K-TRP1::HGR RPT1(WT)::NAT ubp6::KAN*	–	3i
YTS159	*MATa pCUP1-Ub-K-TRP1::HGR rpt1-S164R-T166K::NAT*	–	3i
YTS157	*MATa pCUP1-Ub-K-TRP1::HGR rpt1-S164R-T166K::NAT ubp6::KAN*	–	3i

Supplementary Table 9 | Yeast strains used for canavanine assay.

The strains in this table are isogenic to SUB62 (as above, [6]).

Dashes in the table indicate yeast strains bearing no plasmids.

Yeast Strain	Relevant genotype	Host strain	Figure(s)
SY255c	*MATa ubp6::HIS3*	–	–
ySH19	*MATa ubp6::HIS3 (YCplac33 [CEN4/URA3]) (YEp46Δ [2μ/TRP1])*	SY255c	Supplementary Fig. 7e
ySH20	*MATa ubp6::HIS3 (pJH80: UBP6-WT [CEN4/URA3]) (YEp46Δ [2μ/TRP1])*	SY255c	Supplementary Fig. 7e
ySH51	*MATa ubp6::HIS3 (pySH07: ubp6-UBL [CEN4/URA3]) (YEp46Δ [2μ/TRP1])*	SY255c	Supplementary Fig. 7e
ySH21	*MATa ubp6::HIS3 (pySH01: ubp6-I329A-L330A [CEN4/URA3]) (YEp46Δ [2μ/TRP1])*	SY255c	Supplementary Fig. 7e
ySH24	*MATa ubp6::HIS3 (YCplac33 [CEN4/URA3]) (YEp96: pCUP1-Ub [2μ/TRP1])*	SY255c	Supplementary Fig. 7e
ySH25	*MATa ubp6::HIS3 (pJH80: UBP6-WT [CEN4/URA3]) (YEp96: pCUP1-Ub [2μ/TRP1])*	SY255c	Supplementary Fig. 7e
ySH52	*MATa ubp6::HIS3 (pySH07: ubp6-UBL [CEN4/URA3]) (YEp96: pCUP1-Ub [2μ/TRP1])*	SY255c	Supplementary Fig. 7e
ySH26	*MATa ubp6::HIS3 (pySH01: ubp6-I329A-L330A [CEN4/URA3]) (YEp96: pCUP1-Ub [2μ/TRP1])*	SY255c	Supplementary Fig. 7e
YTS121a	*MATa RPT1(WT)::NAT*	–	3h
YTS115a	*MATa RPT1(WT)::NAT ubp6::KAN*	–	3h
YTS135a	*MATa rpt1-S164R-T166K::NAT*	–	3h
YTS125a	*MATa rpt1-S164R-T166K::NAT ubp6::KAN*	–	3h
YTS129a	*MATa RPT1(WT)::NAT ubp6(KO)-pADH1-Ub::KAN*	–	3h
YTS175a	*MATa rpt1-S164R-T166K::NAT UBP6(WT)-pADH1-Ub::KAN*	–	3h
YTS133a	*MATa rpt1-S164R-T166K::NAT ubp6(KO)-pADH1-Ub::KAN*	–	3h

Supplementary Table 10 | Yeast strains used for *in vivo* Ubp6 noncatalytic assay.

Two strains in this table, SSW32 and SSW72, are isogenic to SUB62 (as above, [6]). All other yeast strains in this table are isogenic to BY4741[14], which has the genotype *MATa his3Δ1 leu2Δ0 met15Δ0 ura3Δ0*.

Dashes in the table indicate yeast strains bearing no plasmids.

Where applicable, superscripted number references the origin of the yeast strain.

Yeast Strain	Relevant genotype	Host strain	Figure(s)
sJH183[10]	*MATa ubp6::HIS3*	–	4e
ySH1	*MATa ubp6::HIS3 (YCplac33 [CEN4/URA3])*	sJH183	4e
ySH2	*MATa ubp6::HIS3 (pJH80: UBP6-WT [CEN4/URA3])*	sJH183	4e
ySH3	*MATa ubp6::HIS3 (pySH01: ubp6-I329A-L330A [CEN4/URA3])*	sJH183	4e
ySH6	*MATa ubp6::HIS3 (pJH81: ubp6-C118A [CEN4/URA3])*	sJH183	4e
ySH7	*MATa ubp6::HIS3 (pySH04: ubp6-C118A-I329A-L330A [CEN4/URA3])*	sJH183	4e
sJH185[10]	*MATa ubp6::HIS3 rpn4::KAN*	–	4e
ySH10	*MATa ubp6::HIS3 rpn4::KAN (YCplac33 CEN4/URA3])*	sJH185	4e
ySH11	*MATa ubp6::HIS3 rpn4::KAN (pJH80: UBP6-WT [CEN4/URA3])*	sJH185	4e
ySH12	*MATa ubp6::HIS3 rpn4::KAN (pySH01: ubp6-I329A-L330A [CEN4/URA3])*	sJH185	4e
ySH15	*MATa ubp6::HIS3 rpn4::KAN (pJH81: ubp6-C118A [CEN4/URA3])*	sJH185	4e
ySH16	*MATa ubp6::HIS3 rpn4::KAN (pySH04: ubp6-C118A-I329A-L330A [CEN4/URA3])*	sJH185	4e
SSW32	*MATa ubp6::NAT rpn4::KAN*	–	4f
SSW272	*MATa rpn4::KAN ubp6::NAT rpt1-S164R-T166K::NAT*	–	4f

Supplementary Table 11 | Reagents used in this study.

Reagent	Source	Reference
Rosetta 2 (DE3) competent cells	EMD Millipore	71397-4
NEB 5-α	NEB	C2988J
IPTG	Gold Biotech	I2481C50
AEBSF (200 mM stock, Water)	Gold Biotech	A5440
Leupeptin	Sigma	L0649
Antipain	Sigma	A6191
Benzamidine hydrochloride	Sigma	199001
Aprotinin	Sigma	A6279
Chymostatin	Sigma	C7268
Pepstatin	Sigma	P5318
Coomassie Plus Bradford Protein Assay	Pierce	PI23236
Ni-NTA agarose	Qiagen	30210
Superdex 200 HiLoad 16/600 column	GE Healthcare	28-9893-35
Glutathione Sepharose 4B resin	GE Healthcare	95017-172
Thrombin protease	Sigma	T7513
Benzamidine Sepharose	GE Healthcare	17-0568-01
BL21 (DE3) competent cells	EMD Millipore	69450-3
cOmplete, Mini, EDTA-free Protease Inhibitor Cocktail Tablets	Roche	11836170001
Amicon Ultra-4 Centrifugal Device, MWCO 30K	EMD Millipore	UFC803024
Superdex 75 10/300 GL column	GE Healthcare	17-5174-01
ATP	Sigma	A3377
M2 anti FLAG agarose	Sigma	A2220
3X FLAG peptide	Sigma	F4799
Superose 6 10/300 GL column	GE Healthcare	17517201
Rabbit IgG resin	MP Biomedicals	855961
DTT	Gold Biotech	DTT50
His-AcTEV protease (10000 U @ 10 U/uL)	Thermo Fisher	12575-023
NeutrAvidin agarose resin	Thermo Fisher	PI29201
UbVS	Boston Biochem	U202
Deuterium oxide D_2O, low paramagnetic	Cambridge Isotope Laboratories	DLM-11-100
ACQUITY UPLC BEH C18, 1.7 µm, 2.1 mm × 5 mm column	Waters	186003975
ACQUITY UPLC BEH C18, 1.8 µm, 1.0 mm × 100 mm column	Waters	186002346
Glu[1]-fibrinopeptide	Sigma	F3261
ovalbumin	Sigma	A5503
Low volume 384-well black flat bottom nonbinding surface microplate	Corning	3820
EnVision plate reader	Perkin Elmer	2103
Ub-AMC	Boston Biochem	U550
suc-LLVY-AMC	Bachem	I-1395
anti-Cdc27 (Clone AF3.1)	Santa Cruz	sc-9972
Protein-A Sepharose 4B fast flow	Sigma	P9424
Creatine kinase	Roche	10127566001
Creatine phosphate	Roche	10621712001
ADP-Potassium Salt (0.25 M stock, 50 mM Tris-HCl [pH7.5], 5 mM $MgCl_2$)	EMD Millipore	117105
Hexokinase (10 U/µL stock, water)	Sigma	H4502
ATPγS (10 mg/mL = 18.28 mM stock, water)	Santa Cruz	sc-214500A
PS-341/Veclade/Bortezomib (2.5 mM stock, DMSO)	ApexBio	A2614
MG-262 (10 mg/mL = 20.4 mM stock, DMSO)	ApexBio	A8179
Epoxomicin (10 mg/mL = 18.03 mM stock, DMSO)	ApexBio	A2606
1,10-phenanthroline (1.5 M stock, DMSO)	Sigma	131377
Lithium acetate	Sigma	L6883
PEG MW 3350	Sigma	P4338
Salmon Sperm DNA (sheared, 10 mg/mL)	Agilent	201190

Supplementary References

1 Aufderheide, A. *et al.* Structural characterization of the interaction of Ubp6 with the 26S proteasome. *Proc Natl Acad Sci U S A* **112**, 8626-8631, doi:10.1073/pnas.1510449112 (2015).

2 Shi, Y. *et al.* Rpn1 provides adjacent receptor sites for substrate binding and deubiquitination by the proteasome. *Science* **351**, doi:10.1126/science.aad9421 (2016).

3 Hu, M. *et al.* Structure and mechanisms of the proteasome-associated deubiquitinating enzyme USP14. *EMBO J* **24**, 3747-3756, doi:10.1038/sj.emboj.7600832 (2005).

4 Eisele, M. R. *et al.* Expanded Coverage of the 26S Proteasome Conformational Landscape Reveals Mechanisms of Peptidase Gating. *Cell Rep* **24**, 1301-1315 e1305, doi:10.1016/j.celrep.2018.07.004 (2018).

5 Beckwith, R., Estrin, E., Worden, E. J. & Martin, A. Reconstitution of the 26S proteasome reveals functional asymmetries in its AAA+ unfoldase. *Nat Struct Mol Biol* **20**, 1164-1172, doi:10.1038/nsmb.2659 (2013).

6 Finley, D., Ozkaynak, E. & Varshavsky, A. The yeast polyubiquitin gene is essential for resistance to high temperatures, starvation, and other stresses. *Cell* **48**, 1035-1046, doi:10.1016/0092-8674(87)90711-2 (1987).

7 Saeki, Y. *et al.* Knocking out ubiquitin proteasome system function in vivo and in vitro with genetically encodable tandem ubiquitin. *Methods Enzymol* **399**, 64-74, doi:10.1016/S0076-6879(05)99005-8 (2005).

8 Houde, D., Berkowitz, S. A. & Engen, J. R. The utility of hydrogen/deuterium exchange mass spectrometry in biopharmaceutical comparability studies. *J Pharm Sci* **100**, 2071-2086, doi:10.1002/jps.22432 (2011).

9 Engen, J. R. & Wales, T. E. Analytical Aspects of Hydrogen Exchange Mass Spectrometry. *Annu Rev Anal Chem (Palo Alto Calif)* **8**, 127-148, doi:10.1146/annurev-anchem-062011-143113 (2015).

10 Hanna, J., Meides, A., Zhang, D. P. & Finley, D. A ubiquitin stress response induces altered proteasome composition. *Cell* **129**, 747-759, doi:10.1016/j.cell.2007.03.042 (2007).

11 Gietz, R. D. & Sugino, A. New yeast-Escherichia coli shuttle vectors constructed with in vitro mutagenized yeast genes lacking six-base pair restriction sites. *Gene* **74**, 527-534 (1988).

12 Hanna, J. *et al.* Deubiquitinating enzyme Ubp6 functions noncatalytically to delay proteasomal degradation. *Cell* **127**, 99-111, doi:10.1016/j.cell.2006.07.038 (2006).

13 Ecker, D. J., Khan, M. I., Marsh, J., Butt, T. R. & Crooke, S. T. Chemical synthesis and expression of a cassette adapted ubiquitin gene. *J Biol Chem* **262**, 3524-3527 (1987).

14 Brachmann, C. B. *et al.* Designer deletion strains derived from Saccharomyces cerevisiae S288C: a useful set of strains and plasmids for PCR-mediated gene disruption and other applications. *Yeast* **14**, 115-132, doi:10.1002/(SICI)1097-0061(19980130)14:2<115::AID-YEA204>3.0.CO;2-2 (1998).
