# Supplementary material for: Allosteric control of Ubp6 and the proteasome via a bidirectional switch
Source: Nat Commun. 2022 Feb 11;13:838. doi: 10.1038/s41467-022-28186-y (PMC8837689; doi:10.1038/s41467-022-28186-y)
Supplement: Supplementary file 2 — Description of Additional Supplementary Files [file 41467_2022_28186_MOESM2_ESM.docx]

**File Name:** Supplementary Data 1

**Description:** This file contains all deuterium uptake values used to generate figures showing Ubp6-dependent deuteration differences within peptides from Rpt1-Rpt6 of purified regulatory particle (Fig. 1d, Supplementary Fig. 5a) and within Ubp6 (Supplementary Fig. 5b). Peptide coverage maps for Rpt1-Rpt6 and Ubp6 are also provided.
