# Supplementary material for: Allosteric control of Ubp6 and the proteasome via a bidirectional switch
Source: Nat Commun. 2022 Feb 11;13:838. doi: 10.1038/s41467-022-28186-y (PMC8837689; doi:10.1038/s41467-022-28186-y)
Supplement: Supplementary file 5 — Source Data Files [file 41467_2022_28186_MOESM5_ESM.zip › 5_Sourcedatafile/Fig2e3g4a4b4c4d.pdf]

Fig. 2e

anti-HA tag

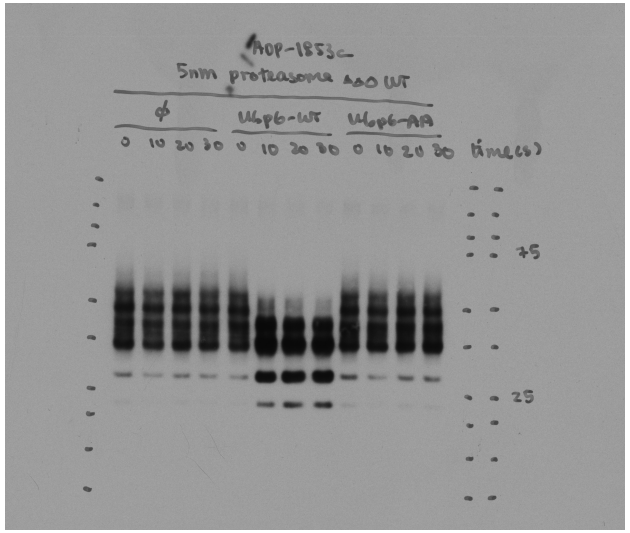

anti-Ubp6

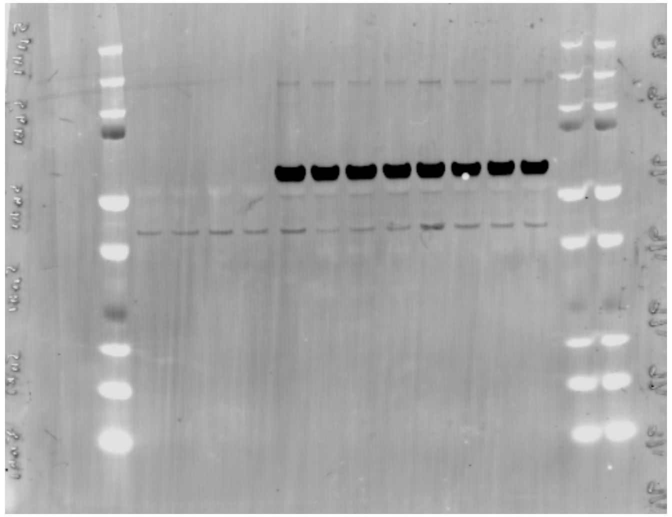

anti-Rpn13

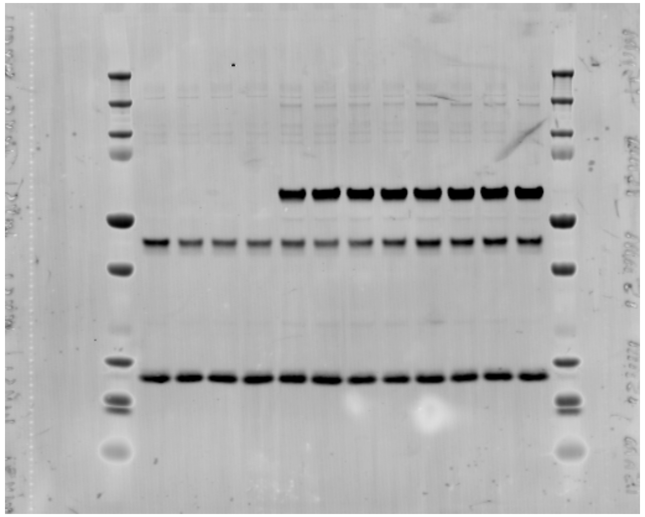

Fig. 3g

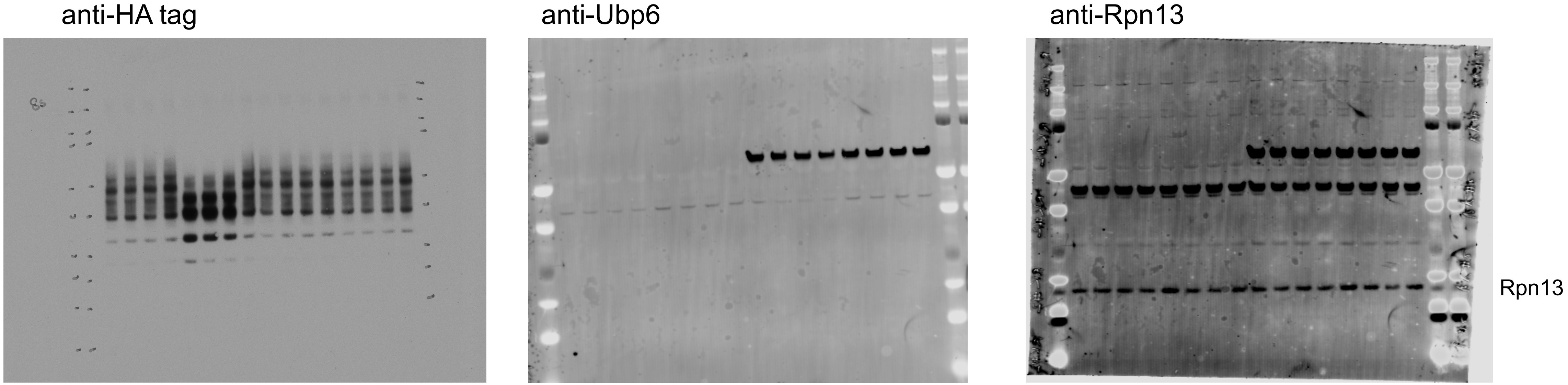

Fig. 4a

anti-HA tag

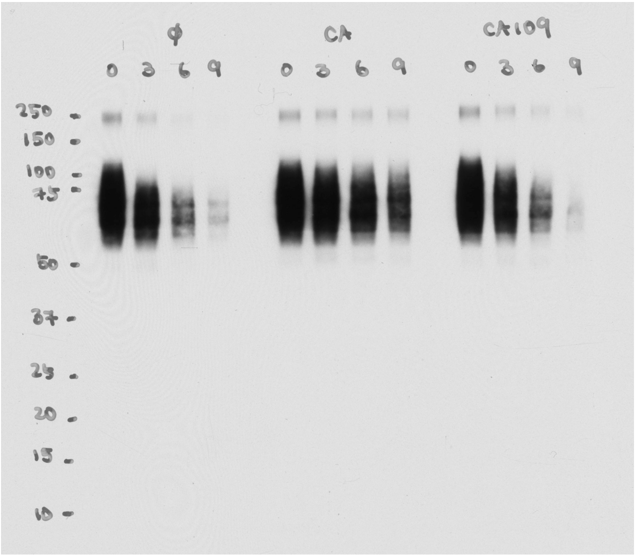

anti-Ubp6, anti-Rpn8

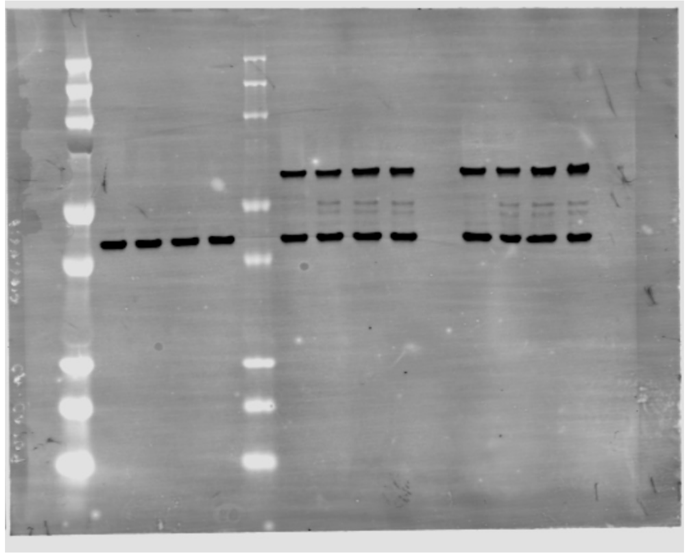

Fig. 4b

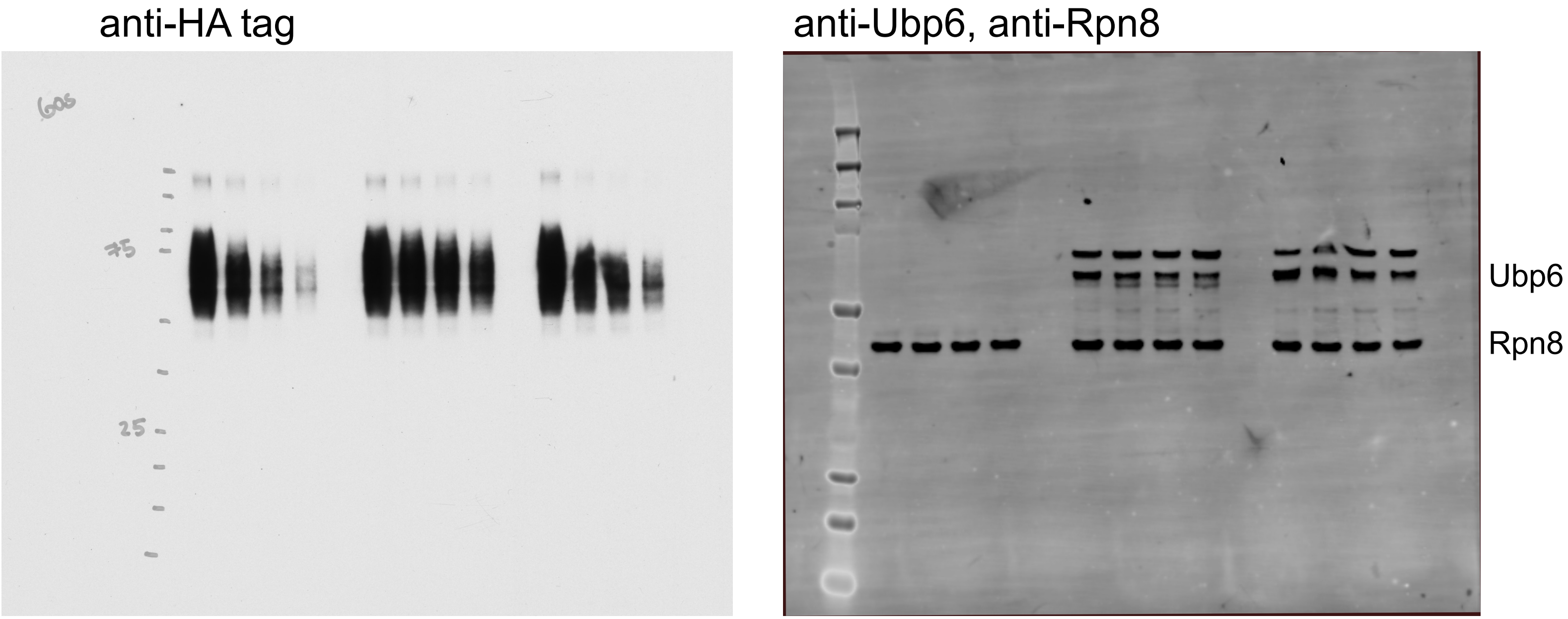

Fig. 4c-4d

anti-HA tag, Fig. 4c

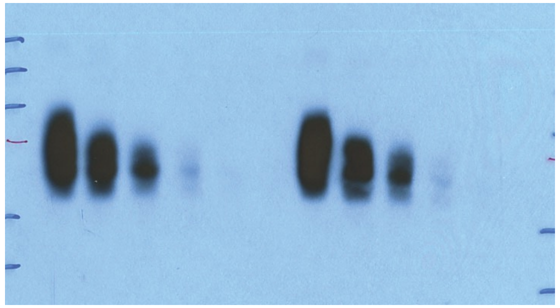

anti-HA tag, Fig. 4d

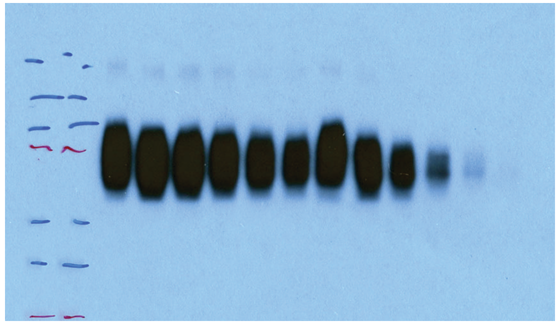

anti-Ubp6, anti-Rpn8

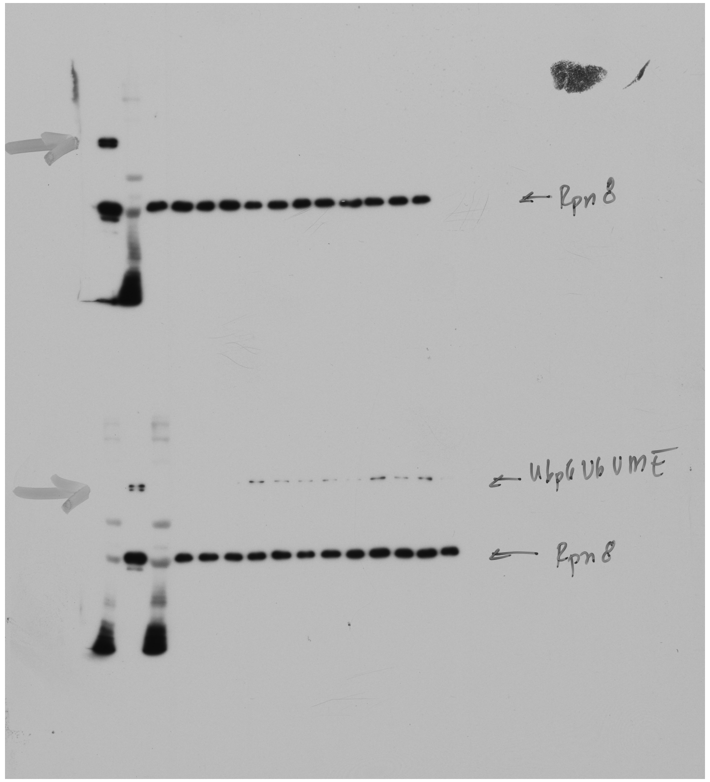

anti-Ubp6, high sensitivity ECL reagents

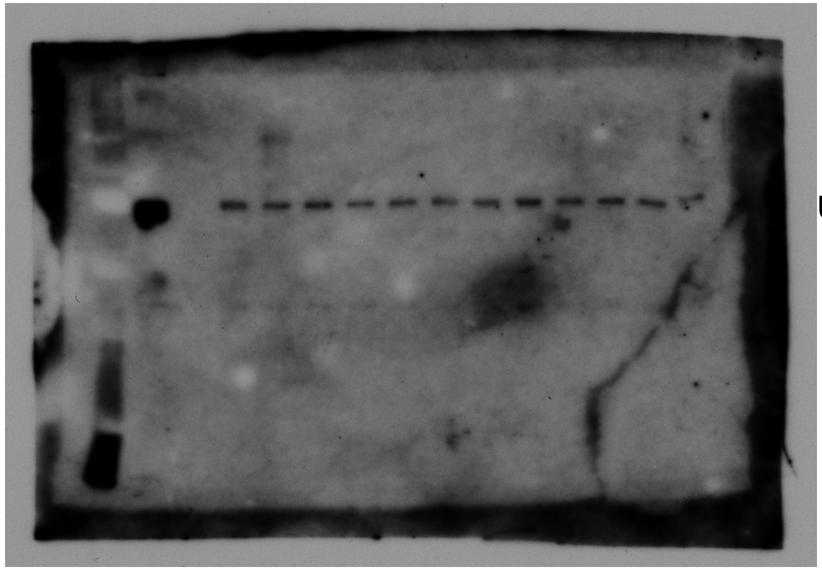

Ubp6-UbVME
